# Robustness, flexibility, and sensitivity in a multifunctional motor control model

**DOI:** 10.1007/s00422-016-0704-8

**Published:** 2016-12-21

**Authors:** David N. Lyttle, Jeffrey P. Gill, Kendrick M. Shaw, Peter J. Thomas, Hillel J. Chiel

**Affiliations:** 1 Department of Mathematics and Biology, Case Western Reserve University, 10900 Euclid Ave., Cleveland, OH 44106 USA; 2 Department of Biology, Case Western Reserve University, 10900 Euclid Ave., Cleveland, OH 44106 USA; 3 Department of Anesthesia, Critical Care, and Pain Medicine, Massachusetts General Hospital, Boston, MA 02114 USA; 4 Department of Mathematics, Applied Mathematics, and Statistics, Case Western Reserve University, 10900 Euclid Ave., Cleveland, OH 44106 USA; 5 Department of Biology, Neurosciences and Biomedical Engineering, Case Western Reserve University, 10900 Euclid Ave., Cleveland, OH 44106 USA

**Keywords:** Adaptive behavior, *Aplysia*, Central pattern generator, Sensory feedback, Heteroclinic channel, Limit cycle, Multistability

## Abstract

Motor systems must adapt to perturbations and changing conditions both within and outside the body. We refer to the ability of a system to maintain performance despite perturbations as “robustness,” and the ability of a system to deploy alternative strategies that improve fitness as “flexibility.” Different classes of pattern-generating circuits yield dynamics with differential sensitivities to perturbations and parameter variation. Depending on the task and the type of perturbation, high sensitivity can either facilitate or hinder robustness and flexibility. Here we explore the role of multiple coexisting oscillatory modes and sensory feedback in allowing multiphasic motor pattern generation to be both robust and flexible. As a concrete example, we focus on a nominal neuromechanical model of triphasic motor patterns in the feeding apparatus of the marine mollusk *Aplysia californica*. We find that the model can operate within two distinct oscillatory modes and that the system exhibits bistability between the two. In the “heteroclinic mode,” higher sensitivity makes the system more robust to changing mechanical loads, but less robust to internal parameter variations. In the “limit cycle mode,” lower sensitivity makes the system more robust to changes in internal parameter values, but less robust to changes in mechanical load. Finally, we show that overall performance on a variable feeding task is improved when the system can flexibly transition between oscillatory modes in response to the changing demands of the task. Thus, our results suggest that the interplay of sensory feedback and multiple oscillatory modes can allow motor systems to be both robust and flexible in a variable environment.

## Introduction

A remarkable feature of animal behavior is the extent to which motor control is both *robust* and *flexible*. Intuitively, both terms refer to the ability of motor systems to adapt to change, either in the environment, in the task, or within the body and nervous system. Adaptability in the face of change is critical for survival, since animals must perform a variety of motor tasks in complex, dynamic environments. Moreover, the internal neural, synaptic, and muscular parameters involved in generating motor patterns typically vary over both short and long timescales due to a diverse range of processes, including growth and development, neuromodulation, learning, noise, injury, and disease. Adapting to change requires that the dynamics of motor systems be selectively *sensitive* to some perturbations but not others, and the types of perturbations that need to be responded to or filtered out change over time.

How can one understand these mechanisms of adaptability? To achieve this goal, we first propose definitions to clarify key terms used in describing the adaptability of motor systems, specifically systems that generate multiphasic motor rhythms, which are ubiquitous throughout biology. We then discuss how sensory feedback and the coexistence of multiple stable dynamical oscillatory modes can facilitate adaptive behavior. Furthermore, we argue that oscillatory modes comprising a stable limit cycle passing close to one or several saddle fixed points (a stable heteroclinic channel) can provide a particularly effective way for a central pattern-generating circuit to incorporate sensory feedback. Finally, we use a minimal neuromechanical model of triphasic motor pattern generation in the *Aplysia* feeding system as a concrete setting in which to demonstrate these ideas.

### Robustness, flexibility, and sensitivity

The challenge of understanding the complex mechanisms involved in the adaptability of motor systems is exacerbated by the lack of a consistent vocabulary. The terms “robustness,” “flexibility,” and “sensitivity” are found throughout the biological and robotic motor control literature (Selverston 2010; Marder and Goaillard 2006), but are often used interchangeably, inconsistently or are left undefined. One factor contributing to this confusion is the interdisciplinary nature of motor control research, which draws upon knowledge and tools from neuroscience, biology, engineering, and applied mathematics. Researchers from different fields often use discipline-specific and nonoverlapping definitions for these terms (Lesne 2008; Kitano 2004; Zhou and Doyle 1998; Meir et al. 2002). Another difficulty stems from the fact that in common usage, “robust” and “flexible” are often used interchangeably. In order to disambiguate these terms, we propose the following definitions. In “Appendix 1” we revisit them in a dynamical systems framework.

We define the *robustness* of a motor system with respect to a perturbation of its state variables or internal parameters as the ability of the motor system to maintain its fitness in performing a task in the presence of the perturbation. Evaluating the robustness of a system requires a measure of task-specific fitness.^1^ Given such a measure, we can quantify the robustness of a motor system by introducing a perturbation and comparing the performance of the perturbed system to that of the unperturbed system. A more robust system will exhibit a less dramatic degradation in performance in response to the perturbation than a system that is less robust. For example, if task performance is measured in terms of walking speed during locomotion, animals that are robust with respect to the addition of a load will show only a modest reduction in walking speed when the load is applied. In a general setting this notion of robustness parallels the notion of homeostasis (Nijhout et al. 2004; Nijhout and Reed 2014) which can be interpreted mathematically in terms of invariances (or approximate invariances) of functionals of vector fields to changes in parameters (Golubitsky and Stewart 2016).

We define *flexibility* as the ability of a motor system to deploy alternative strategies in order to perform better on different tasks or to respond to changes in the task requirements. A motor system that produces the same behavioral output, even if better strategies are available, is less flexible than one that can select an appropriate strategy from a larger repertoire of possible behaviors. Like robustness, flexibility is evaluated with respect to a measure of behavioral performance. However, flexibility allows motor systems to improve fitness by adjusting behavioral output in response to perturbations, whereas robustness buffers against reductions in fitness by filtering out perturbations. Note that the ability to change strategies alone is an insufficient criteria for flexibility—our definition also requires that the change in strategy produces an improvement in performance over the original strategy. Thus, for example, in traveling through rough, sloping, and irregular terrain, there may be times when crawling, or using one’s hands as well as one’s legs to find footholds and handholds may be needed in addition to regular walking movements, and these variations in locomotion strategy enhance the overall fitness (i.e., the distance covered). Since the human locomotory system is capable of improving its performance by changing strategies in response to varying conditions, we say that it is a flexible motor system.

Another important concept that may help us characterize robustness and flexibility is *sensitivity*. Sensitivity describes the extent to which the dynamics of a motor system change in response to perturbations or parameter variations. Unlike robustness and flexibility, which are measured in terms of behavioral fitness, sensitivity refers only to the responsiveness of a system to perturbations. Sensitivity to perturbations can be either beneficial, detrimental, or neutral with respect to task performance, and sensitivity refers only to the magnitude of the response to a perturbation, rather than its effect on fitness. In some contexts, high sensitivity may impair robustness by amplifying the effects of small, task-irrelevant perturbations or by causing a system to fail if a parameter falls outside a narrow tolerable range. However, in other contexts, high sensitivity (particularly to sensory inputs) can allow the dynamics of a motor system to adapt to changes in the environment, thereby facilitating flexibility. For example, in the rodent whisking system, high sensitivity may be detrimental to robustness during free whisking, but once the whiskers encounter an obstacle or environmental boundary, higher sensitivity may be required for accurately extracting the contours of the environment (Mitchinson et al. 2007; Hartmann 2001).

### Dynamical architectures, sensory integration, and multifunctionality

**Fig. 1:**
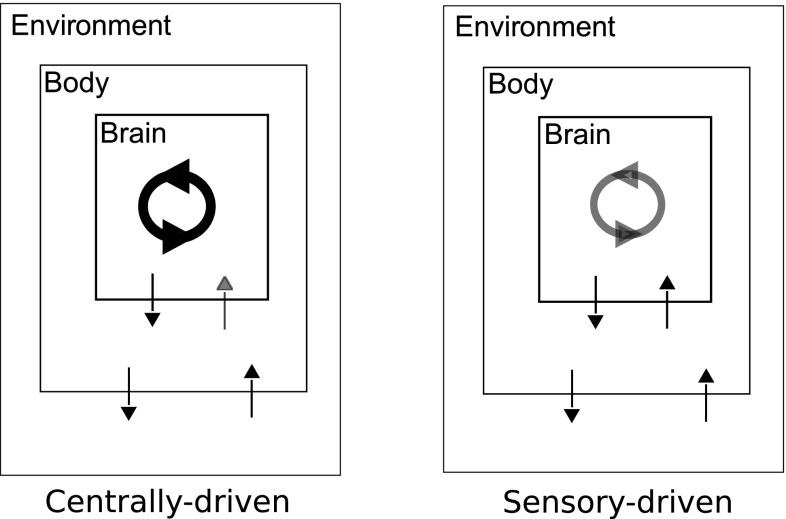
Dynamical architectures for motor pattern generation differ in their responsiveness to sensory input. *Left* At one extreme, the endogenous oscillatory dynamics generated within the nervous system (*cycling arrows*) drive the musculature (*brain* $\rightarrow$ *body*) and produce behavior (*body* $\rightarrow$ *environment*). Mechanical perturbations (*environment* $\rightarrow$ *body*) may have only weak effects on the central neural dynamics since they are insensitive to sensory inputs (*body* $\rightarrow$ *brain*). This architecture facilitates robustness in behaviors for which the sensitivity to internal and external perturbations should be low, but can fail during behaviors that require high sensitivity to sensory inputs. *Right* In contrast, in a sensory-driven pattern generator, the neural dynamics depend upon appropriately timed sensory inputs to progress through the cycle, rather than being driven by an endogenous pattern generator. This architecture can facilitate robustness in behaviors that require the timing of motor outputs to change in response to environmental conditions, since the sensitivity to sensory input is high, but can fail for behaviors where high sensitivity to sensory inputs or their absence is detrimental

Different classes of dynamical systems have been proposed to describe motor pattern generation. These classes differ significantly in their sensitivity to proprioceptive inputs and parameter variations (Fig. 1). Recently, several authors have explored a class of dynamical systems called a “stable heteroclinic channel” (SHC) (Afraimovich et al. 2004a, b). In an SHC, a limit cycle passes in close proximity to one or more saddle points. The presence of these fixed points near the cycle creates localized regions where the dynamics become slow, thereby allowing sensory feedback to slow or even stop the progress of the cycle in specific phases, by either moving the trajectory closer to the fixed points or forcing a collision with a boundary in phase space (Shaw et al. 2012, 2015). Consequently, SHC-based models can produce reliable oscillations in the absence of sensory feedback while still retaining sensitivity to sensory feedback.

Many motor systems are multifunctional, and the various tasks they perform may have different requirements for sensitivity to sensory feedback. Such motor systems must be flexible to meet these requirements. How can a multifunctional motor pattern generator flexibly adjust its sensitivity in response to changing task demands? One possibility is that sensory feedback can facilitate multifunctionality by triggering a switch between coexisting stable attractors, or by pushing a system through a bifurcation to produce qualitatively different motor outputs.

Here we propose that an SHC-based dynamical architecture can facilitate flexibility in a multifunctional motor system by allowing for sensory feedback-triggered switching between low-sensitivity and high-sensitivity dynamics in response to varying task demands.

Many rhythmic behaviors require an alternation between a loaded phase (e.g., during stance, when a leg generates ground reaction forces) and an unloaded phase (e.g., during the swing phase of locomotion). These alternations emerge from the limits on lengths of limbs and are completely general. Because of its experimental tractability, we focus on a specific example of rhythmic behavior, a neuromechanical model of feeding behaviors in the marine mollusk *Aplysia californica*, which must also alternate between a loaded phase (when food is being drawn into the feeding apparatus) and an unloaded phase (when the feeding apparatus is repositioned to grasp food).

**Fig. 2:**
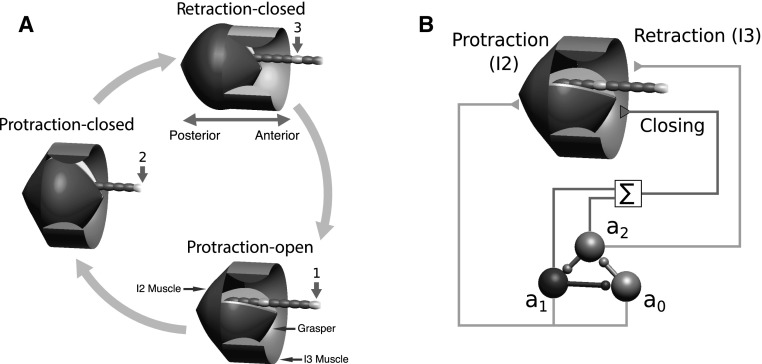
Phases of ingestive behavior and neural pool-muscle relationships in the model. **a** Ingestive behaviors in the model can be divided into three major phases, depending on the direction of movement and state (open or closed) of the grasper: protraction while open (*1*), protraction while closed (*2*), and retraction (*3*). The grasper (*red*) is moved anteriorly (to the *right*) by the contraction of the sheet-like protractor muscle (I2; *blue*). The grasper is moved posteriorly (to the *left*) by the contraction of the ring-like retractor muscle (I3; *yellow*). A section of the retractor muscles has been cut away so that the grasper is visible. The *green strand* is seaweed, with the *arrows* indicating seaweed movement. **b** The protractor muscle is activated by the $a_0$ and $a_1$ neural pools, and the retractor muscle is activated by the $a_2$ neural pool. The grasper is closed if the sum $a_1 + a_2$ exceeds a specified threshold and open otherwise. The protraction-open phase corresponds to $a_0>\text {max}\{a_1,a_2\}$, the protraction-closed phase corresponds to $a_1>\text {max}\{a_0,a_2\}$, and the retraction-closed phase corresponds to $a_2>\text {max}\{a_0,a_1\}$. As each neural pool activates in turn, it inhibits the neural pool preceding it in the sequence. This, in combination with weak endogenous excitation in each neural pool, is sufficient to create periodic activation that drives ingestive behavior. Figure adapted from Shaw et al. (2015) (color figure online)

Using our model, we demonstrate that, for a range of parameters, two oscillatory modes coexist. We then show that one mode is more robust to parametric perturbations of sensory pathways, whereas the other is more robust to perturbations of the mechanical load. We also show that the application of an external seaweed load can induce transitions between the two oscillatory modes. Finally, we study the performance of this model using a feeding task with time-varying requirements, and we show that the best performance occurs when the system can flexibly transition between oscillatory modes as needed to facilitate either foraging for food (biting) or ingesting grasped food (swallowing). Thus, we demonstrate one possible mechanism by which even very simple pattern-generating circuits produce behavior that is both robust and flexible.

## Methods and model description

### Mathematical framework

To understand how animals produce robust and flexible behavior in a variable environment, we must consider how the nervous system interacts with the body and how the system as a whole interacts with the outside world (Chiel and Beer 1997). Therefore, our model incorporates both neural and biomechanical components, with bidirectional coupling between the two, as well as interactions between the biomechanical variables and the outside world in the form of externally applied loads. The neural activity drives the behavior of the musculature, and sensory feedback, including proprioceptive feedback about the state of the muscles, in turn modifies the neural activity.

Systems of this general form can be described mathematically using the following set of equations adapted from Shaw et al. (2015):

1 $$\begin{aligned} \frac{\mathrm{d}\mathbf {a}}{\mathrm{d}t}&= f(\mathbf {a}) + g(\mathbf {a},\mathbf {x}), \end{aligned}$$

2 $$\begin{aligned} \frac{\mathrm{d}\mathbf {x}}{\mathrm{d}t}&= h(\mathbf {a}, \mathbf {x}) + l(\mathbf {x}). \end{aligned}$$

Here $\mathbf {a}$ is a vector of neural activation variables, and $\mathbf {x}$ is a vector of mechanical state variables representing both the body and the outside world. The intrinsic dynamics of the central pattern generator are described by $f(\mathbf {a})$, and sensory feedback from the mechanical variables perturbs these dynamics through the term $g(\mathbf {a},\mathbf {x})$. The neurally driven dynamics of the mechanical variables are given by $h(\mathbf {a}, \mathbf {x})$, which are also subject to external mechanical loads as described by the term $l(\mathbf {x})$.

### Model overview

Here we focus on one specific instance of a multiphasic pattern-generating neuromechanical system, namely a model of feeding behaviors in the herbivorous marine mollusk *Aplysia californica*. The mathematical framework described here, however, is sufficiently general to be applied to a broad range of multiphasic pattern generators. We focus on *Aplysia* because it is an experimentally tractable model organism, making experimental tests of our model’s predictions feasible.

*Aplysia californica* feeds on seaweed. The animal uses an organ located in its head called the buccal mass, or feeding apparatus, to pull seaweed into its mouth during feeding. A grasper structure (the radula-odontophore) within the buccal mass is capable of moving forward toward the jaws (protraction), closing on food, moving backward toward the esophagus (retraction), and opening to release the food. These movements repeat cyclically during ingestive behavior. The grasper will frequently close before protraction ends (Cullins et al. 2015).

In the model, as a consequence of the dynamics that we will describe below, ingestive behaviors can be divided into three major phases: (1) forward movement of the grasper while open (protraction-open), (2) forward movement of the grasper while closed (protraction-closed), and (3) backward movement of the grasper while closed (retraction-closed). This sequence is illustrated schematically in Fig. 2a. Triphasic motor patterns of this sort are found in a number of vertebrate and invertebrate systems (Marder 2000; Rubin et al. 2009).

In our model, we simulate the neural pattern generator that drives this behavior using a simplified firing rate description of three mutually inhibitory neural pools. These pools activate in a fixed sequence and correspond to the three phases of swallowing. Since the muscles respond slowly, the grasper position lags behind the neural signal activating the muscles. Thus, the first and second neural pools ($a_0$ and $a_1$) activate the protraction muscle (the I2 muscle), and their activity induces the protraction-open and protraction-closed phases, respectively. The third pool ($a_2$) activates the retractor muscle (the I3 muscle), and its activity induces the retraction-closed phase. Biologically, these three pools represent the activity of (potentially overlapping) collections of motor neurons and interneurons. For example, the $a_0$ neural pool includes the B31, B32, B61, B62, and B63 neurons, the $a_1$ neural pool includes these same neurons with the addition of the B8 motor neurons (which receives slow excitation from B34), and the $a_2$ neural pool includes B64, B3, B6, B9, and the B8 motor neurons (which are excited by B64). The grasper opens or closes when the summed activity of the second and third pools crosses a predetermined threshold (here set to 0.5). The relationship between the neural pools and the different phases of the swallowing cycle is illustrated in schematic form in Fig. 2b.

Our model also includes two muscles that work antagonistically to move the grasper: a protractor muscle representing I2 and a retractor muscle representing I3, both of which have unique nonlinear length-tension curves. Muscles operate at slower timescales than neurons, and thus, muscle activation depends on a low-pass-filtered version of the neural activity. We make the assumption, based on experimental observations, that the biomechanical variables are overdamped, which allows us to simplify the equations for the movement of the grasper in response to muscular forces.

The governing equations and details of each aspect of our model are described below. Tables of state variables and model parameters are provided in “Appendix 2.” Simulation codes for this paper were written in C$++$ and Mathematica and are available on GitHub (https://github.com/CWRUChielLab/Lyttle_et_al_2017_code).

### Neural model

The dynamics of the neural state variables **a** are a combination of the intrinsic dynamics of the circuit, $f(\mathbf {a})$, and sensory inputs, $g(\mathbf {a},\mathbf {x})$. For simplicity, we assume that these effects sum linearly (see Eq. 1).

#### Intrinsic circuit dynamics

Our model nervous system consists of a sequence of three neural pools, connected such that each pool is inhibited by the next one in the sequence (see Fig. 2b). A system of Lotka–Volterra equations describes their intrinsic dynamics:

3 $$\begin{aligned} f_0(\mathbf {a})&= \frac{1}{\tau _\text {a}}(a_0 (1 - a_0 - \gamma a_1) + \mu ), \end{aligned}$$

4 $$\begin{aligned} f_1(\mathbf {a})&= \frac{1}{\tau _\text {a}}(a_1 (1 - a_1 - \gamma a_2) + \mu ), \end{aligned}$$

5 $$\begin{aligned} f_2(\mathbf {a})&= \frac{1}{\tau _\text {a}}(a_2 (1 - a_2 - \gamma a_0) + \mu ). \end{aligned}$$

This Lotka–Volterra-based model of the neural dynamics is motivated by the observation that the motor patterns in *Aplysia* are driven by groups of self-exciting cells inhibiting other groups of self-exciting cells (Kabotyanski et al. 1993). Equations of this type have previously been used to model inhibitory neural networks in several contexts, including the generation of motor behaviors in mollusks (Rabinovich et al. 2001; Varona et al. 2002; Levi et al. 2004).

Here $\tau _\text {a}$ is the time constant of the neural dynamics, $\gamma$ controls the strength of the inhibitory connections between pools, and $\mu$ is a small positive parameter representing endogenous excitation. Neural excitability in the *Aplysia* cerebral and buccal ganglia (represented here by $\mu$) can be influenced by factors such as temperature, or the action of neuromodulators representing arousal. For example, the release of serotonin by the metacerebral cell modulates excitability in buccal ganglion cells, increases the frequency of rhythmic motor patterns, and has been linked to food-induced arousal states in vivo (Kupfermann et al. 1979). When $\mu = 0$, the intrinsic neural dynamics contain a stable heteroclinic cycle with an infinite period. For positive $\mu$, the isolated neural dynamics contain a stable limit cycle with a finite period that passes near a sequence of three fixed points, referred to as a stable heteroclinic channel (SHC) (Rabinovich et al. 2008). Increasing $\mu$ increases the distance between the limit cycle and the fixed points and speeds up the oscillation frequency of the limit cycle.

#### Sensory feedback

We use a simple description of the sensory input to the nervous system which is the sum of two components: the first is proprioceptive feedback dependent on the position of the grasper, and the second is tactile and chemical sensory information signaling the presence or absence of seaweed in the buccal cavity. The proprioceptive input takes the form of a linear function of the grasper position, given by

$$\begin{aligned} \epsilon (x_\text {r} - S_i) \sigma _i, \end{aligned}$$

where $x_\text {r} \in [0,1]$ is the position of the grasper. $S_i$ determines the position where the proprioceptive input to the *i*th neural pool is zero, $\epsilon$ is a constant parameter that controls the overall strength of the input, and $\sigma _i \in \{-1,1\}$ is the direction of proprioceptive feedback for the *i*th neural pool. These parameters are set such that the $a_0$ protraction pool is excited when the grasper is retracted (when it is time to protract) and inhibited when it is protracted (when it is time to retract); the $a_1$ protraction and closing pool and, more strongly, the $a_2$ retraction and closing pool are excited when the grasper is protracted (when it is time to close and retract) and inhibited when the grasper is retracted (when it is time to open and protract).

In later simulations, we challenge the neuromechanical model with a more realistic feeding task. In these simulations, we allow for seaweed to sometimes be absent from the buccal cavity. We introduce a second type of sensory feedback, *K*, that provides the neuromechanical system with feedback about the presence or absence of seaweed (in real *Aplysia*, this sense is facilitated by mechano- and chemosensors). We assume this seaweed-triggered input is additive with the proprioceptive input previously described. When seaweed is present in the buccal cavity, $K= -\kappa \mu /\tau _a$ acts to inhibit all three neural pools equally; when seaweed is absent, $K=0$. The parameter $\kappa \in [0,1]$ represents the strength of the seaweed-triggered inhibition relative to the endogenous excitation $\mu$. We model this seaweed-triggered input as an inhibitory input so that the model will operate in the heteroclinic mode and exhibit more swallow-like behavior.

Thus, the full expression for the sensory input to the $i\text {th}$ neural pool is given by

6 $$\begin{aligned} g_i (x_\text {r}) = \epsilon (x_\text {r} - S_i) \sigma _i + K. \end{aligned}$$

Unlike the general form presented in Eq. 1, this does not depend on the states of the neural pools themselves.

#### Boundary conditions

The neural activation variables $a_{i}$ model the normalized firing rates of the neural pools, where $a_{i} = 0$ indicates that the neurons are inactive, and $a_{i} = 1$ indicates that the neurons are firing at maximum frequency. We assume that when excitatory or inhibitory sensory feedback is received by neural pools that are, respectively, already firing at maximum frequency or are inactive, the neural activation variables are unaffected.

To enforce these assumptions, we apply strict rectifying boundary conditions to the neural state variables to prevent them from dropping below zero or exceeding one. Specifically, whenever $a_{i} = 0$ and $f_{i}(\mathbf {a}) + g_{i}(\mathbf {a}, \mathbf {x})< 0$, we set $\frac{\mathrm{d} a_{i}}{\mathrm{d}t} = 0$, and when $a_{i} = 1$ and $f_{i}(\mathbf {a}) + g_{i}(\mathbf {a},\mathbf {x}) > 0$, we set $\frac{\mathrm{d} a_{i}}{\mathrm{d}t} = 0$. The presence of such rectifying boundary conditions creates the possibility of nonsmooth border collisions under variation of parameters, which can lead to a rich array of dynamical effects (Simpson 2016).

### Biomechanical model

We model the movement of the grasper by calculating the forces generated by two sets of muscles acting antagonistically to move the grasper forward toward the jaws (protraction, mediated by contraction of the I2 muscle) or backward toward the esophagus (retraction, mediated by contraction of the I3 muscle; see Fig. 2).

Muscle activation is slow relative to the timescale of the neural dynamics (Yu et al. 1999) and thus is modeled as a low-pass-filtered version of the neural inputs:

7 $$\begin{aligned} \frac{\mathrm{d}u_0}{\mathrm{d}t}&= \frac{1}{\tau _\text {m}}((a_0 + a_1)u_\text {max} - u_0), \end{aligned}$$

8 $$\begin{aligned} \frac{\mathrm{d}u_1}{\mathrm{d}t}&= \frac{1}{\tau _\text {m}}(a_2 u_\text {max} - u_1). \end{aligned}$$

Here $u_{0}$ and $u_{1}$ are the activation variables for the protraction and retraction muscles, respectively, $\tau _{m}$ is the time constant of muscle activation (with $\tau _{a} \ll \tau _{m}$), and $u_\text {max}$ is the maximum muscle activation.

We model the length-tension curves for each muscle using a cubic polynomial:

9 $$\begin{aligned} \phi (x) = -\frac{3\sqrt{3}}{2} x (x - 1) (x + 1). \end{aligned}$$

This form of the length-tension curve produces zero force when the muscles have length zero or one (representing the maximum length). The scaling factor $3\sqrt{3}/2$ normalizes the function to a peak value of 1.

The net force exerted by the muscles is given by the difference in protractor and retractor muscle forces:

10 $$\begin{aligned} F_\text {musc} = \phi \left( \frac{c_0 - x_\text {r}}{w_0}\right) u_0 - \phi \left( \frac{c_1 - x_\text {r}}{w_1}\right) u_1. \end{aligned}$$

Here $c_{i}$ and $w_{i}$ describe the mechanical (length-tension) properties of each muscle. Net positive force moves the grasper toward the jaws (protraction), and net negative force moves the grasper toward the esophagus (retraction). The signs on the terms are determined by the direction of force exerted by the muscles: the I2 muscle (first term) protracts the grasper when it contracts, whereas the I3 muscle (second term) retracts the grasper. The parameters $c_0$, $w_0$, $c_1$, and $w_1$ were chosen based on biomechanical studies (Yu et al. 1999; Neustadter et al. 2002; Sutton et al. 2004a; Novakovic et al. 2006) so that the force exerted by the posteriorly positioned sheet-like I2 muscle is maximal when the grasper is fully retracted and zero when the grasper is fully protracted; the force exerted by the anteriorly positioned ring-like I3 muscle is maximal when the grasper is near the middle of its range and decreases as it is further protracted or retracted.

Coupling to the outside world is simulated by applying a mechanical load to the grasper when it is in contact with the seaweed. Specifically, when the grasper is closed on seaweed, the seaweed applies a resisting force, $F_\text {sw}$, that counters inward movement during retraction, and which may vary in time.

Based on experimental observations of *Aplysia* grasper movements, we assume that the dynamics governing grasper motion are overdamped and thus use the following simplified equation for the movement of the grasper [see the appendix in Shaw et al. (2015) for a derivation]:

11 $$\begin{aligned} \frac{\mathrm{d}x_\text {r}}{\mathrm{d}t} = {\left\{ \begin{array}{ll} \frac{F_\text {musc} + F_\text {sw}}{b_\text {r}} &{} \text {if closed on seaweed}, \\ \frac{F_\text {musc}}{b_\text {r}} &{} \text {otherwise}. \\ \end{array}\right. } \end{aligned}$$

where $b_\text {r}$ is a damping constant and the condition $a_1 + a_2 > 0.5$ corresponds to the grasper being closed; the grasper can only be closed on seaweed if seaweed is present in the buccal cavity.

### Seaweed movement

When the grasper is closed on seaweed, the grasper and seaweed move together (i.e., there is no slippage between the grasper and seaweed). When the grasper is open, we assume the seaweed does not move, even if $F_\text {sw} > 0$. This assumption is supported by recent work in *Aplysia* demonstrating that the jaws are capable of holding seaweed while the grasper is open, preventing it from moving outward while under tension (McManus et al. 2014). Thus, the movement of seaweed is governed by the following equation:

12 $$\begin{aligned} \frac{\mathrm{d}x_\text {sw}}{\mathrm{d}t} = {\left\{ \begin{array}{ll} \frac{F_\text {musc} + F_\text {sw}}{b_\text {r}} &{} \text {if closed on seaweed}, \\ 0 &{} \text {otherwise}. \\ \end{array}\right. } \end{aligned}$$

### Simulated feeding tasks

#### Continuous-swallowing task

For several of our simulations, we assess the performance of the model using a pure swallowing task, assuming an infinite strip of seaweed. In some of these simulations, the seaweed force $F_\text {sw}$ is assumed to be constant over the duration of the simulation, but in other simulations, we explore the effects of allowing the strength of the force to vary over time. For all simulations involving the continuous-swallowing task, we do not include the seaweed-triggered inhibition component of the sensory feedback, since seaweed is always present in the buccal cavity (essentially setting $\kappa = 0$).

#### Forage-and-feed task

For the simulations used at the end of Results section (Sect. 3.4), we assess the performance of the model using a forage-and-feed task that requires both biting and swallowing. In the biting (“forage”) phase of this task, seaweed is absent from the buccal cavity, and $K = 0$. The system can grasp food and switch to the swallowing (“feed”) phase according to the following rule: on every closure, the grasper either fails to contact food or succeeds in grasping a finite length of seaweed with some probability, here taken to be $p = 0.1$. If the grasper succeeds in making contact with seaweed, the task switches to the swallowing phase, and $K = -\kappa \mu /\tau _a$. The system must successfully swallow a fixed length of seaweed (here set to $L = 0.5$ seaweed units), after which it returns to the biting phase and attempts to grasp another piece.

### Quantifying robustness of the neuromechanical feeding system

Our dynamical systems framework for analysis of robustness and flexibility in motor pattern generation leads naturally to empirically testable hypotheses involving experimentally measurable quantities (Appendix 1). In this subsection we show how the general framework presented there applies to this particular system. For the neuromechanical *Aplysia* feeding model performing the continuous-swallowing task, with an idealized fitness measured by the net seaweed consumption rate, the “fitness” depends only on the net displacement of the grasper position during the grasper-closed component of the trajectory, and the period of the limit cycle. Let $\Delta x_0$ be the net change in grasper position while the grasper is closed during one cycle under a nominal constant load $\lambda _0$. If we change the load from $\lambda _0$ to $\lambda _0+\epsilon$, then we see empirically a roughly linear shift in $\Delta x$, i.e.,

13 $$\begin{aligned} \Delta x (\epsilon )\approx \Delta x_0 + \epsilon \Delta x_1, \end{aligned}$$

for small $\epsilon$. We also see empirically that there is an approximately linear change in the timing, i.e., in the duration *T* of a complete trajectory,

14 $$\begin{aligned} T(\epsilon )\approx T_0 + \epsilon T_1, \end{aligned}$$

for small $\epsilon$.^2^ Taking the average seaweed intake rate to be our measure of fitness,

15 $$\begin{aligned} S=\frac{\Delta x}{T} \end{aligned}$$

we can expand *S* for small $\epsilon$. Define $S_0=\Delta x_0/T_0$. Then

16 $$\begin{aligned} S(\epsilon )&=\frac{\Delta x(\epsilon )}{T(\epsilon )} =\frac{\Delta x_0+\epsilon \Delta x_1+O(\epsilon ^2)}{T_0+\epsilon T_1+O(\epsilon ^2)}\nonumber \\&=S_0 + \epsilon S_0 \left( \frac{\Delta x_1}{\Delta x_0} - \frac{T_1}{T_0} \right) +O(\epsilon ^2), \text { as }\epsilon \rightarrow 0.\nonumber \\ \end{aligned}$$

Therefore, the relative shift in intake rate per change in load, for small $\epsilon$, can be written in terms of the relative changes in timing and grasper displacement,

17 $$\begin{aligned} \frac{1}{S_0}\left. \frac{\partial S}{\partial \lambda }\right| _{\lambda _0}&\approx \frac{1}{\epsilon }\frac{S(\epsilon )-S_0}{S_0}\nonumber \\&= \frac{\Delta x_1}{\Delta x_0} - \frac{T_1}{T_0} + O(\epsilon ),\text { as }\epsilon \rightarrow 0. \end{aligned}$$

We note that Eq. 17 gives two terms that can in principle be measured and compared empirically from experimental data, regardless of the internal details of the system. When a load is applied, what is the shift in the open duration, the closed duration, and the open movement and closed movement of the grasper (and seaweed)? Is it true that on a swallow-by-swallow basis, the distance retracted while closed is strongly correlated with the duration of the entire motion (or the duration of the protraction-closed phase)? Although these are empirical questions, we show how to address them in the model (see below, Fig. 8).

## Results

### Multiple modes and bistability

**Fig. 3:**
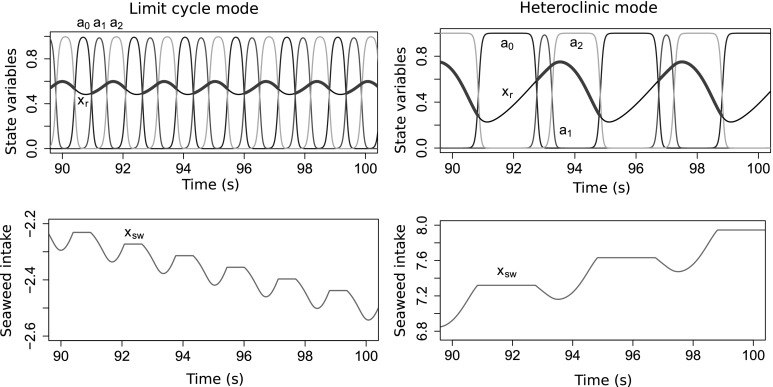
Model of the *Aplysia* feeding apparatus has two coexisting stable oscillatory modes that exhibit distinct behaviors: the limit cycle mode (*left*) and heteroclinic mode (*right*). In both cases, the task is continuous-swallowing, the endogenous excitation $\mu = 10^{-5}$, and the mechanical load $F_{sw} = 0$ is constant. The different behaviors are produced by using different initial conditions for the neural state variables: ($a_{0}(0)$, $a_{1}(0)$, $a_{2}(0)$) is ($1 - 10^{-9}$, $10^{-9}$, $10^{-9}$) for the heteroclinic mode and (0.2, 0.4, 0.7) for the limit cycle mode. *Top panels* Trajectories of the three neural state variables $a_{0}$, $a_{1}$, and $a_{2}$ (*blue*, *red*, and *yellow*, respectively), and the position of the grasper (*black* when open, *thick dark green* when closed). In the limit cycle mode, the neural dynamics are relatively insensitive to sensory feedback, so the durations of the three phases of the motor pattern are short and approximately equal. Since the muscles are slow to respond to changes in the neural variables ($\tau _m \gg \tau _a$), rapid neural cycling does not allow adequate time for the muscles to develop strong forces or to fully relax, causing the antagonistic muscles to be tonically moderately activated, and thus movement is limited. In contrast, in the heteroclinic mode, the neural dynamics are sensitive to and asymmetrically slowed by proprioceptive feedback. The protraction-open ($a_0$, *blue*) and retraction-closed ($a_2$, *yellow*) phases last longer, resulting in a longer cycle period overall and greater range of motion. *Bottom panels* Movement of the seaweed. In the limit cycle mode, since the durations of the protraction-closed ($a_1$, *red*) and retraction-closed ($a_2$, *yellow*) phases are approximately equal, the grasper pushes seaweed out more than it pulls seaweed in each cycle, resulting in a net loss of food. In contrast, in the heteroclinic mode, the retraction-closed phase is extended, and seaweed is consumed (color figure online)

When subjected to a constant load, our model can produce two qualitatively different triphasic oscillatory behaviors that exhibit different levels of sensitivity to sensory feedback. In this section we demonstrate that these two modes can coexist, and thus the system exhibits bistability. Furthermore, we show that the likelihood of the system utilizing one oscillatory mode or the other depends on the strength of the endogenous excitation ($\mu$) relative to that of the sensory feedback ($g(\mathbf x )$).

In Shaw et al. (2015) we showed that the neuromechanical model described in Sects. 2.3–2.5 3 can produce the two oscillatory modes for different values of the endogenous excitation parameter $\mu$, which we will now briefly summarize.

In the first oscillatory mode, which is more prevalent when the endogenous excitation parameter $\mu$ is small, the system has high sensitivity to sensory inputs, and its dynamics are primarily sensory-driven. The limit cycle of the coupled neuromechanical system projected onto the neural state variables ($a_0$, $a_1$, $a_2$) passes close to one or more of the fixed points of the isolated (uncoupled) neural dynamics ${\dot{\mathbf{a}}} = f(\mathbf {a})$. These orbits are sensitive to perturbations from sensory feedback, which can push the trajectory into or out of regions of phase space near those fixed points in which the velocity is very small. This can result in sensory feedback extending or truncating specific phases of the motor pattern. We refer to this oscillatory mode as the “heteroclinic mode,” since the neural dynamics are strongly influenced by the stable heteroclinic channel structure.

In the second oscillatory mode, which is more prevalent when the endogenous excitation parameter $\mu$ is large, the system is insensitive to sensory inputs, and its dynamics are primarily centrally driven. The limit cycle components ($a_0$, $a_1$, $a_2$) of the coupled system remain relatively far from the fixed points of the isolated neural dynamics, and consequently the dynamics of the coupled system are much less sensitive to mechanical load. We refer to this oscillatory mode as the “limit cycle mode,” since the neural dynamics are more similar to that of a classic stable limit cycle.

In Shaw et al. (2015) we showed that a sudden transition between the two modes occurs as the parameter $\mu$ *varies* with initial conditions *fixed* (a bifurcation). In this paper, we report the coexistence of the two oscillatory modes for *fixed* values of $\mu$ in a narrow, intermediate range with *varying* initial conditions (bistability).

To illustrate the bistability of the model and qualitative differences between modes, we first show example trajectories obtained by running the model with the same parameter values and different initial conditions (Fig. 3). The limit cycle mode (left) is incapable of ingesting food because the neural pools cycle too rapidly for the muscles, which act like low-pass filters, to keep pace. Consequently, the grasper does not move much. In contrast, seaweed is ingested successfully in the heteroclinic mode (right) because the neural dynamics temporarily slow during certain phases of the cycle, allowing the muscles to catch up.

**Fig. 4:**
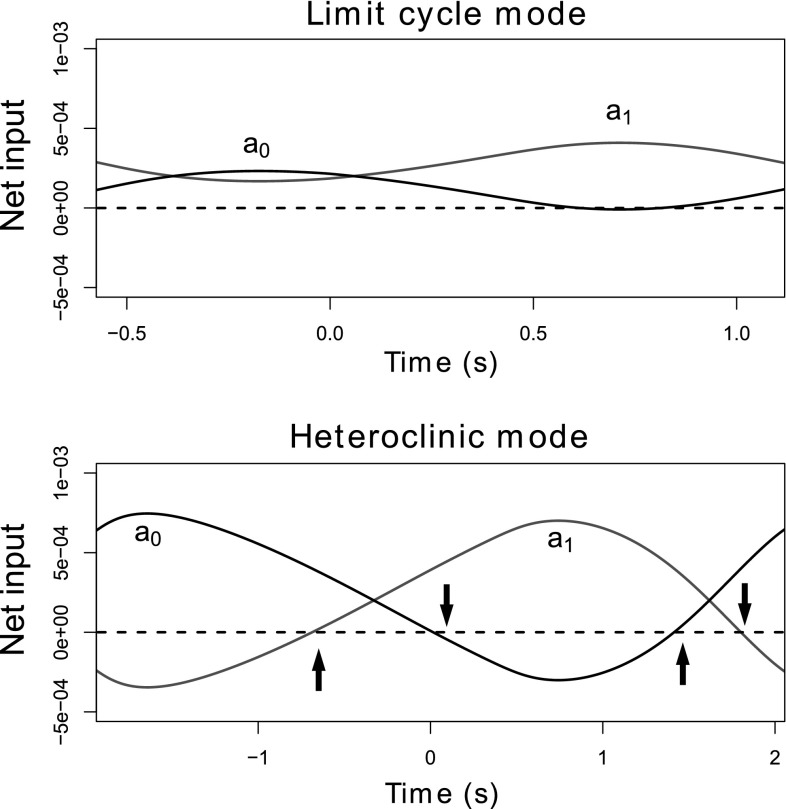
Mechanism distinguishing the two oscillatory modes. The key difference is whether the neural variables collide with the boundaries. This can occur when the proprioceptive feedback $g_i(x_\text {r})$ overcomes the endogenous excitation $\mu /\tau _\text {a}$, such that the net input is negative. Plots show the total inputs, $g_i(x_\text {r}) + \mu /\tau _\text {a}$, to the protraction-open ($a_{0}$, *blue*) and protraction-closed ($a_{1}$, *red*) neural pools over one complete cycle in the limit cycle mode (*top*) and in the heteroclinic mode (*bottom*). Here the beginning of the cycle is defined as the onset of the $a_{0}$ neural pool, and time $t=0$ corresponds to the closing of the grasper. Parameters and initial conditions as in Fig. 3. The *dashed lines* indicate zero net input. *Bottom* In the heteroclinic mode, the net input can become negative due to the proprioceptive inputs overcoming the endogenous excitation (*downward arrows*). If this net negative input is sufficiently strong, and the neural state variable receiving it is sufficiently close to zero, the trajectory of the neural variables will collide with the boundary $a_i = 0$. Collision with the boundary stops the cycling of the neural variables, which does not resume until the grasper reaches the appropriate position, releasing the neural pool from inhibitory proprioceptive input (*upward arrows*). *Top* Such boundary collisions do not occur in the limit cycle mode. Consequently, in this mode the neural cycle progresses almost independently of the state of the biomechanical variables. Note that we did not plot the total input to the $a_\text {2}$ pool because it never overcomes the endogenous excitation in either oscillatory mode (color figure online)

**Fig. 5:**
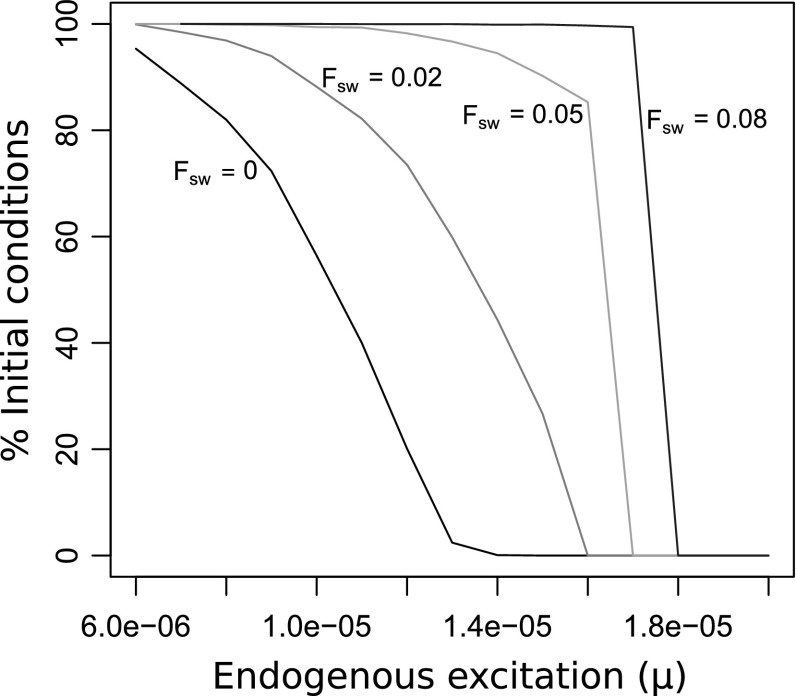
Heteroclinic mode is more common than the limit cycle mode when endogenous excitation is weak and load is large. The percentage of initial conditions sampled for the neural pool variables converging to the heteroclinic mode is plotted on the *vertical axis* (standard errors are approximately 0.5$\%$ and are not shown). *Each line* corresponds to a different value of a fixed resisting force. For each combination of $\mu$ and $F_\text {sw}$, 10,000 independent simulations were performed, each with initial conditions for the three neural state variables sampled independently and uniformly from within the unit cube. Initial conditions for the other state variables were always the same: $x_\text {r}(0)=0.5, u_0(0)=0$, and $u_1(0)=0$. For sufficiently small $\mu$, virtually all initial conditions converge to the heteroclinic mode. As $\mu$ increases, this proportion drops, and for sufficiently large $\mu$, almost all initial conditions converge to the limit cycle mode. In contrast, as $F_\text {sw}$ increases, this proportion increases as long as $\mu$ is not too great. This is explained by the mechanism of competing endogenous excitation and inhibitory sensory feedback (see Fig. 4): since larger force evokes greater sensory feedback, net input to a neural pool is more likely to become negative if $F_\text {sw}$ is large and $\mu$ is small

The mechanism responsible for temporarily slowing the neural variables in the heteroclinic mode is inhibitory sensory feedback overcoming endogenous neural excitation and forcing the neural trajectory to collide with a boundary, $a_i=0$, completely silencing one neural pool. This process is illustrated in Fig. 4, which shows that the total input, $g_i(x_\text {r}) + \mu /\tau _\text {a}$, for two neural pools becomes sufficiently negative during phases of the pattern in the heteroclinic mode to halt neural cycling, allowing the muscles to catch up; this never happens in the limit cycle mode.^4^

Intrinsic excitability can vary within a neural circuit, and this shifts the availability of the different dynamical modes. Since the model only enters the heteroclinic mode when the sensory input is able to overcome the endogenous excitation, the system is more likely to be in the heteroclinic mode (i.e., its basin of attraction is larger) when the endogenous excitation parameter $\mu$ is small and the load $F_\text {sw}$, which affects sensory feedback, is large (see Fig. 5). Conversely, the basin of attraction for the heteroclinic mode shrinks as $\mu$ increases or as $F_\text {sw}$ decreases.

Thus, we see that the system exhibits bistability across a range of endogenous excitation ($\mu$) and seaweed force ($F_{sw}$) values, and that the two modes differ in their sensitivities to sensory input. Moreover, increasing the endogenous excitation makes the system more likely to be in the limit cycle mode, whereas increasing the force (which creates stronger proprioceptive feedback) makes the system more likely to be in the heteroclinic mode. This suggests a possible mechanism for flexibility in the model, if the system can transition into the heteroclinic mode as a way of dealing with increased seaweed force during swallowing. We discuss the functional implications of this bistability-induced flexibility in Sect. 3.4.

### Sensitivity and robustness of the two modes with respect to sensory parameters

**Fig. 6:**
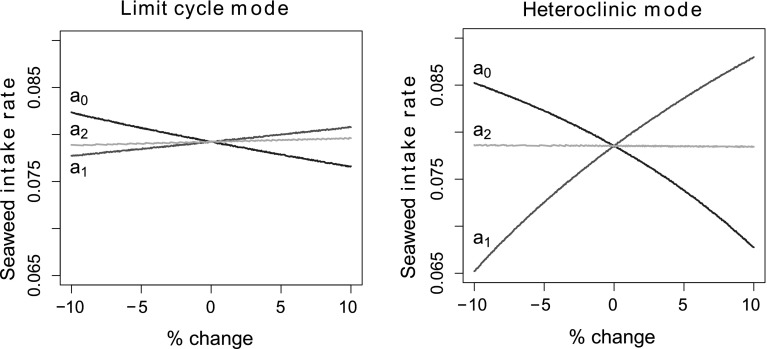
Limit cycle mode is more robust to changes in sensory input parameters than the heteroclinic mode. Plots show the rate of seaweed intake in the continuous-swallowing task with $F_\text {sw} = 0$ across a range of values (between $-10\%$ and $+10\%$ of their default values) for the sensory input parameters $S_{i}$. The *blue*, *red*, and *yellow* lines represent the effect of varying the input parameters to the $a_{0}$, $a_{1}$, and $a_{2}$ neural pools, respectively. *Right* The slopes of the red and blue lines are significantly steeper in the heteroclinic mode case, indicating that in this mode, the performance of the model is more strongly affected by small changes in parameter values. *Left* In comparison, the performance of the limit cycle mode changes much less in response to varying sensory input parameters. The limit cycle mode is more robust in the sense that it is less susceptible to potentially deleterious changes in the parameters. Here the limit cycle mode has been tuned to have comparable performance to that of the heteroclinic mode when the parameters are unperturbed (color figure online)

A key difference between the oscillatory modes stems from the different roles played by sensory feedback. We predicted that the limit cycle mode (centrally driven) would be more robust to perturbations of sensory parameters than the heteroclinic mode (sensory-driven). To test this, we focused on parameters involved in the proprioceptive feedback terms of the model equations, $g_i(x_\text {r})$ (Eq. 6). Specifically, we perturbed the parameters $S_{i}$ governing how proprioceptive feedback is integrated into the neural pools and measured how these changes affected performance of the system (the rate of seaweed consumption).^5^ As we expected, we found that the centrally driven limit cycle mode^6^ is more robust to parametric perturbations of the proprioceptive feedback pathways than the sensory-driven heteroclinic mode (see Fig. 6).

### Sensitivity and robustness of the two oscillatory modes to changes in mechanical load

In addition to internal perturbations, motor systems must be robust to changes in external conditions, such as load. To compare the robustness of the oscillatory modes to changing load, we first compare their responses to a fixed load increase for the continuous-swallowing task. Next, we compare their feeding performance when subjected to loads that varied randomly in time.

**Fig. 7:**
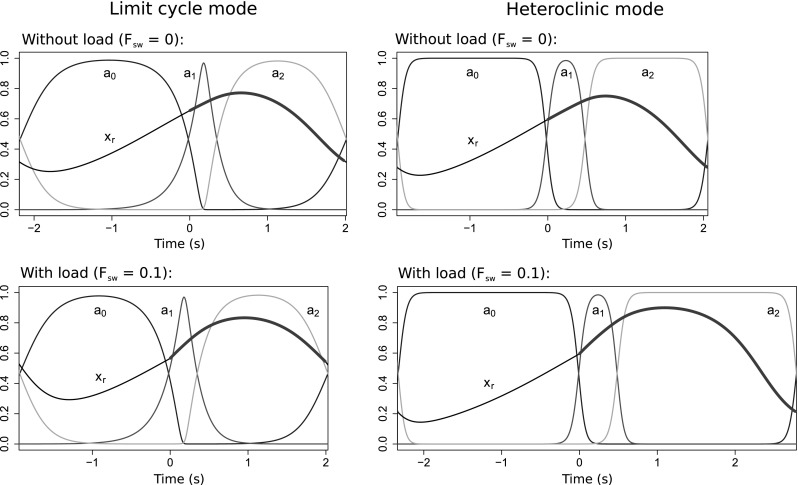
Heteroclinic mode is more responsive to changes in mechanical load than the tuned limit cycle mode. *All panels* show the time courses of the neural activity variables and position of the grasper for one cycle in the continuous-swallowing task (time axes have the same scale in all panels; see Fig. 3 for color key). *Right* In the heteroclinic mode, the motor pattern adapts to an increase in load (from $F_\text {sw} = 0$ to $F_\text {sw} = 0.1$) by lengthening the durations of the protraction-open and retraction-closed phases. *Left* In contrast, in the limit cycle mode, the motor pattern does not change significantly in response to the same load increase (color figure online)

We find that the heteroclinic mode is more sensitive to changes in load than is the limit cycle mode. Figure 7 shows that, in the heteroclinic mode, the neuromechanical system adapts to a load increase by lengthening certain phases of the motor pattern, which allows the muscles to build up more force to compensate for the increased load. In contrast, in the limit cycle mode, the motor output is largely unchanged.^7^

**Fig. 8:**
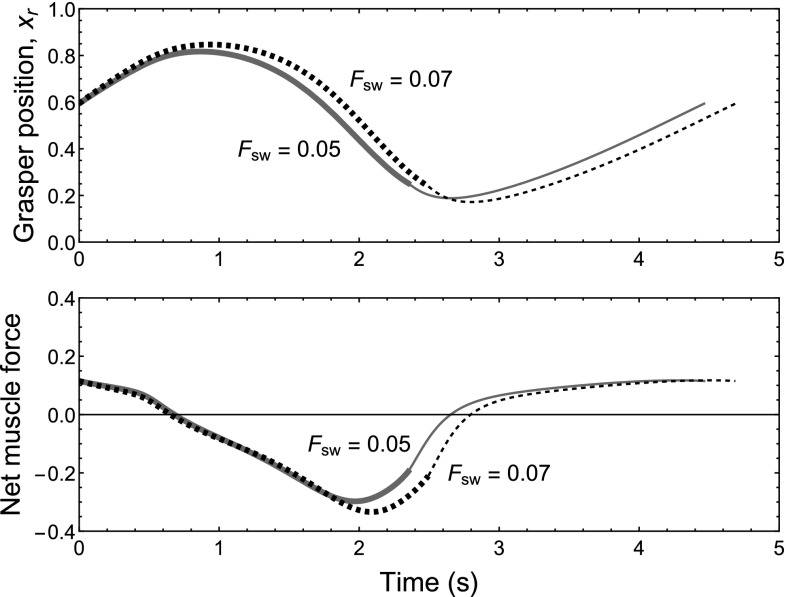
In the heteroclinic mode the system compensates for a load increase by pulling *longer* and *stronger* during the retraction phase of the motion. *Plots* show the change in grasper position (*top*) and net muscle force applied (*bottom*; Eq. 10) before and after a 40% increase in load while in the heteroclinic mode ($\mu =10^{-5}$). *Solid green lines* load condition $F_\text {sw}=0.05$. *Dashed black lines* load condition $F_\text {sw}=0.07$. *Thick segments* grasper closed on seaweed. *Thin segments* grasper open. Under the higher load, grasper displacement during the closed phase (and hence seaweed displacement) *increases by 4%*, i.e., the grasper takes in more seaweed per cycle. This is achieved by a 25% increase in the integrated net force (inward) applied to the seaweed during the grasper-closed period. At the same time the cycle time *lengthens by 5%*, reducing the fitness measure (length of seaweed consumed per second). The net effect on fitness is a decrease of 1%, an order of magnitude smaller than the 40% percent load increase (color figure online)

**Fig. 9:**
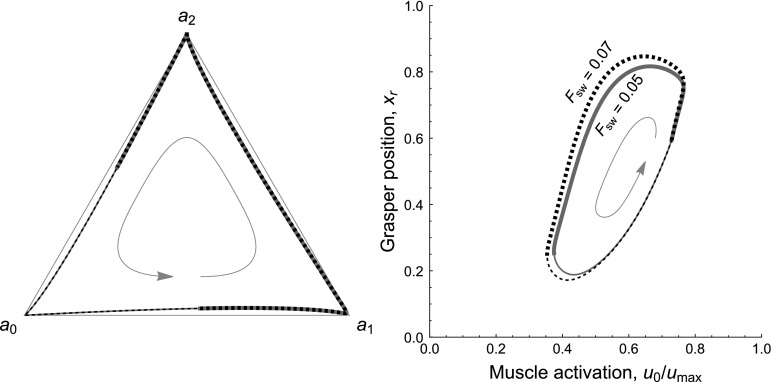
Robustness to load perturbations in the heteroclinic mode is achieved through adjustments in the timing of neural activation, but not the trajectory paths of the neural variables through phase space. Panels show planar projections of the neural variables (*left*) and the body variables (*right*) for two trajectories operating in the heteroclinic mode ($\mu =10^{-5}$) under different loads (as in Fig. 8). *Solid green lines* load condition $F_\text {sw}=0.05$. *Dashed black lines* load condition $F_\text {sw}=0.07$. *Thick line segments* grasper closed on seaweed. *Thin line segments* grasper open. See footnote 8 for description of projections. *Arrows* indicate the direction of cycling. *Left* In a planar projection of the neural variables, the paths of the two trajectories are indistinguishable, and only their timing may differ. *Right* A planar projection of the body variables shows that the heteroclinic mode responds to load by increasing retractor muscle activation (*left on the x-axis*) and retracting farther (*down on the y-axis*). Since the neural variables follow the same path, the difference in their activity driving the changes in the body variables is in the timing of transitions between the neural fixed points (color figure online)

How the heteroclinic mode compensates for load and maintains performance in the continuous-swallowing task is illustrated in Figs. 8 and 9.

Figure 8 shows time plots of the grasper position and the net force applied to the grasper by the muscles before and after a 40% increase in load. Unexpectedly, with higher load, the amount of seaweed ingested each cycle *increases by 4%*. This is made possible by a 25% increase in the integrated net force (inward) generated by the muscles during the grasper-closed period. At the same time, the total duration of one cycle *lengthens by 5%*, reducing the fitness measure (length of seaweed consumed per second). The net effect on fitness is a decrease of 1%, an order of magnitude smaller than the 40% percent load increase. Thus, in the heteroclinic mode, the system compensates for the load increase by adjusting the retraction phase of the motion to pull *longer and stronger* on the seaweed stipe.

With reference to Eq. 17, the most elementary observation concerning the mechanism of robustness shown here is that $\Delta x_1$ and $T_1$ have the same sign (i.e., both the amount of seaweed ingested and the total cycle duration increased). In contrast, for the untuned limit cycle case, $\Delta x_1$ and $T_1$ have opposite signs (not shown), so performance goes from bad to significantly worse; the system does not “adapt” to the load.

Visualizing the trajectories of the state variables in phase space allows us to investigate the effects of load on the trajectory paths, independent of time. In general, a six-dimensional, nonlinear, piecewise-smooth dynamical model can be difficult to analyze. Fortunately, our model admits a convenient pair of two-dimensional projections^8^ that significantly aid in qualitative analysis, as we now illustrate.

For the same simulations presented in Fig. 8, we plot a projection of the neural variables in Fig. 9, left. The heteroclinic mode might have compensated for load by, for example, decreasing the maximum activation of some of the neural pools, or allowing more than one neural pool to be partially active simultaneously. However, we see in the phase space plot that the low-load and high-load trajectories are indistinguishable, suggesting that the pattern of neural activity never changes.

In Fig. 9, right, we plot a projection of the mechanical variables. In this representation, differences in the trajectories of the low-load (solid green) and high-load (dashed black) orbits are very clear. When load is increased, the grasper is pulled to a more protracted position shortly after closing on seaweed (start of thick lines at tail of arrow), the retractor muscle is activated more strongly (displacement to the left), and the grasper moves to a more retracted position before opening (end of thick lines). The trajectories converge while the grasper is open.

Since the path of the trajectory in the neural projection is not altered by a small load increase (Fig. 9, left), but muscle activation and grasper retraction amplitude is changed (Fig. 9, right), this suggests that the *locus of control* allowing the heteroclinic mode to maintain fitness when challenged with load is primarily in the *timing of transitions* from one neural fixed point to the next.

**Fig. 10:**
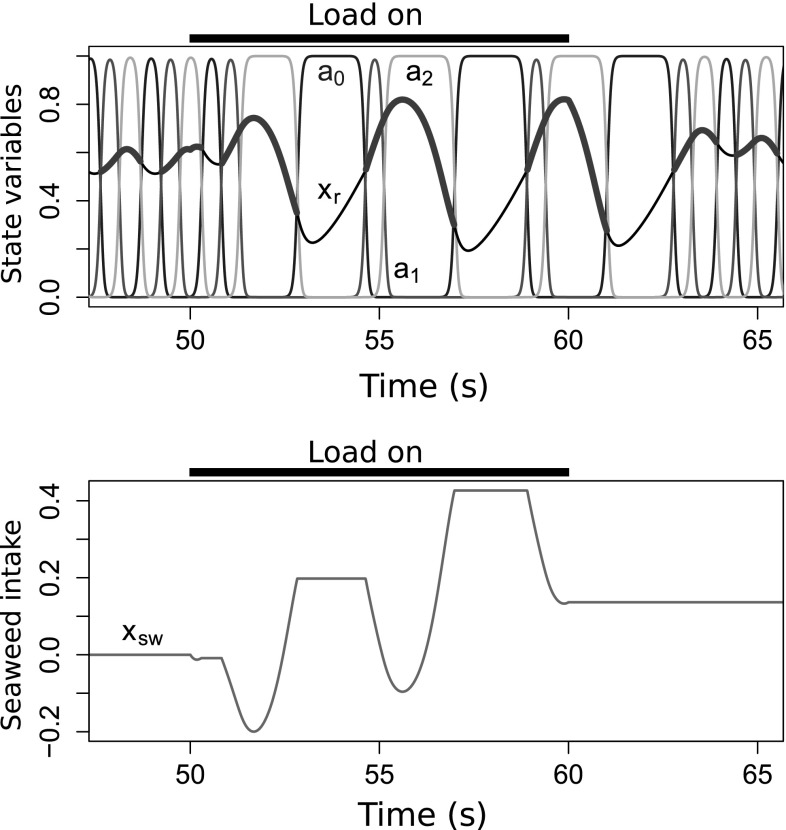
Transitions between oscillatory modes can be induced solely by load. In this example of the forage-and-feed task, $\mu = 1.6 \times 10^{-5}$, $\kappa = 0$, all parameters other than load are constant, and seaweed was placed in the buccal cavity for a predetermined period of time. The system starts in the limit cycle mode without seaweed, biting rapidly. As soon as the grasper closes on the force-loaded seaweed (“*Load on*”; $F_\text {sw} = 0.09$), it is pulled forward by the force. One cycle later, the system transitions to the heteroclinic mode because the increased protraction triggers increased inhibitory proprioceptive feedback to the protraction-open neural pool ($a_0$, *blue*) that overcomes endogenous excitation (see Fig. 4), allowing the retraction-closed neural pool ($a_2$, *yellow*) to remain active longer during the following retraction phase. This results in greater retractor muscle force and an enhanced retraction of the grasper, allowing the system to successfully overcome the load on the seaweed and pull it inward. The enhanced retraction triggers increased inhibitory proprioceptive feedback to the protraction-closed neural pool ($a_1$, *red*) that again overcomes endogenous excitation, and the following protraction-open phase of the grasper movement is further enhanced. This cycling in the heteroclinic mode continues until the seaweed is removed (or consumed), after which the system transitions back to the limit cycle mode. With $\kappa = 0$, this transition is not guaranteed to occur for all seaweed forces and is less likely to occur for weak forces. See Fig. 3 for full color key (color figure online)

How does the robustness of the heteroclinic mode compare to the limit cycle mode when the load varies? We measured the net seaweed intake over the course of an extended run for the continuous-swallowing task, during which both the heteroclinic mode and the tuned limit cycle mode were subjected to equivalent load switching paradigms. The seaweed load varied according to the following rule: on each closure, the load changed with some probability (here set to 0.4). Each time the load changed, the value of the seaweed force was randomly reset to a value chosen from a uniform distribution on the interval $0 \le F_\text {sw} \le 0.1$. We computed the mean seaweed intake rate over the course of a 300-second simulation, repeated for 1000 independent runs, for both the tuned limit cycle mode and the heteroclinic mode. We found that the average seaweed intake rate was significantly higher (two-tailed *t* test, ${p} < 10^{-16}$) in the heteroclinic mode (mean = 0.75, standard deviation = 0.0006 lengths of seaweed per second) than in the limit cycle mode (mean = 0.47, standard deviation = 0.006 lengths of seaweed per second). This shows that the heteroclinic mode adapts better to changing load conditions.

### Multifunctionality and flexibility in feeding performance

The flexibility of feeding in *Aplysia* is due to its ability to switch from attempts to grasp food (biting) to consuming food that has been grasped (swallowing). These two behaviors are required for successful feeding and have different requirements for robustness. Can the two oscillatory modes of the model be combined flexibly to generate higher overall fitness? As shown in Sects. 3.2–3.3, the model can produce rhythmic motor patterns in two distinct dynamical modes, both with their own advantages: one is more robust to changes in the internal parameters (the limit cycle mode; see Fig. 6) and one is more robust to changes in the external load (the heteroclinic mode; see Fig. 7). Furthermore, as shown in Sect. 3.1, the system is *multistable*, and, when a load is time-invariant, the two modes coexist for a range of values of the endogenous excitation parameter $\mu$ and the seaweed force $F_\text {sw}$ (see Figs. 3, 5). We investigated the possible functional advantages of sensory-driven switching between these two modes by testing the model against the forage-and-feed task (see Sect. 2.6.2), which requires both bite-like and swallow-like behaviors.

Without further modification of the model, can it switch between the two modes? Figure 10 shows that, even without seaweed-triggered inhibition ($\kappa = 0$), contact with force-loaded seaweed can trigger a transition from the limit cycle mode to the heteroclinic mode, and that when the seaweed is removed or consumed, the system will transition back to the limit cycle mode. Such a situation can occur in *Aplysia* when a freely biting animal succeeds in grasping seaweed and transitions from biting to swallowing (Kupfermann 1974; Weiss et al. 1986).

**Fig. 11:**
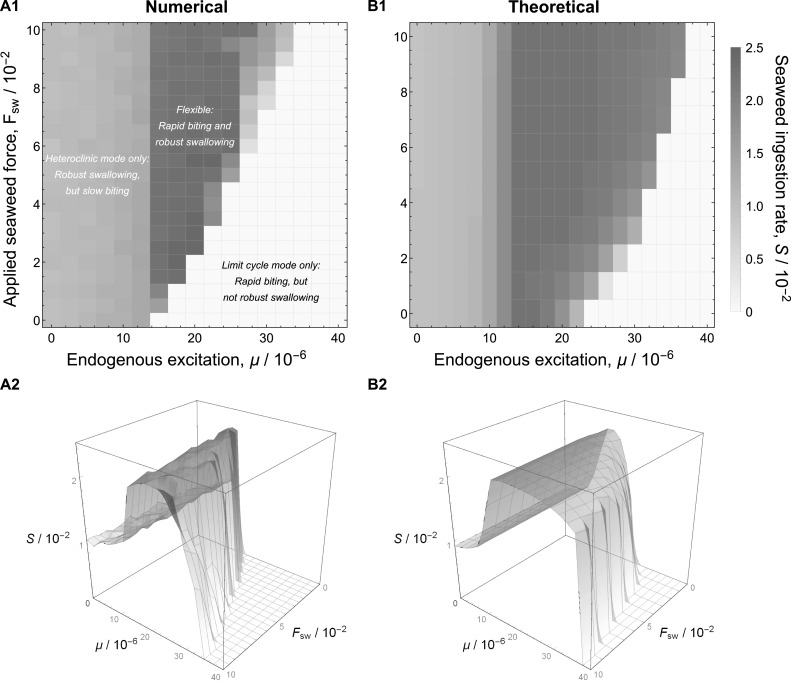
Fitness is maximized for the forage-and-feed task when the system flexibly switches between oscillatory modes. *Left* Performance of the model (seaweed intake rate, *S*) on the forage-and-feed task as a function of endogenous excitation ($\mu$) and seaweed force ($F_\text {sw}$), presented as a heat map (**a1**) and a 3D surface plot (**a2**). Here performance is measured in terms of mean seaweed intake over a 300-second trial, averaged over 500 trials for each set of parameter values. The strength of the seaweed-triggered inhibition is fixed at $\kappa =0.5$. Across nearly the entire range of forces explored, mean seaweed intake peaks at a nonzero $\mu$ value, forming the ridge that runs down the $F_\text {sw}$-axis of the plot. This ridge corresponds to parameter combinations that allow for flexible switching between oscillatory modes. In contrast, when $\mu$ is too small, the system never leaves the heteroclinic mode, and performance is moderate because biting is slow. Similarly, when $\mu$ is too large, the seaweed-triggered inhibition is too weak to induce a switch into the heteroclinic mode, and the system operates only in the limit cycle mode, which fails to swallow seaweed. *Right* Predicted performance of the model on the forage-and-feed task based on measurements from the continuous-swallowing task, presented as a heat map (*b1*) and a 3D surface plot (**b2**). Equation 20 (see “Appendix 3”; $L=0.5$, $\kappa =0.5$, $p=0.1$ to match simulations) approximates the mean seaweed intake rate and captures the key qualitative features of the numerically computed plots (*left*), particularly the region of enhanced performance at intermediate values of $\mu$. Note that the range of $\mu$ values over which this improvement occurs matches well with the numerical plots

The model performs best on the forage-and-feed task when it *flexibly* transitions back and forth between oscillatory modes, depending on the presence of seaweed. Figures 11a1 (a heat map representation) and 11a2 (a 3D surface plot representation) plot the seaweed intake rate (*S*) of the model under these conditions for various combinations of endogenous excitation ($\mu$) and seaweed force ($F_\text {sw}$). When seaweed is absent in the forage-and-feed task, performance is enhanced if the model operates in the limit cycle mode for biting, since cycling is more rapid in this mode and seaweed is likely to be captured sooner. Once seaweed is captured, performance is enhanced if the model switches to the heteroclinic mode for swallowing, since only in this mode can it robustly respond to load. The ridge that runs along the length of the $F_\text {sw}$-axis in Fig. 11a2, which corresponds to the green region in Fig. 11a1, shows that, across nearly all force values, there is a range of intermediate $\mu$ values where performance is maximized by the ability of the system to switch flexibly between oscillatory modes.

#### Mechanism of improved performance in the intermediate-$\mu$ regime

In general, the ability to transition between oscillatory modes affords a neuromechanical system a functional advantage on complex tasks involving abrupt changes in behavioral requirements, because each mode may be best suited to one set of requirements. In our model, the heteroclinic mode is better for swallowing, since the system can cope with the increased load by increasing the duration of retraction. In contrast, the limit cycle mode is better suited for the biting component of the task, since the higher oscillation frequency provides the model with more frequent opportunities to successfully grasp seaweed. Thus, the best performance occurs for values of $\mu$ large enough for the biting frequency to be high in the absence of seaweed (when the seaweed-triggered inhibition is inactive), but small enough for the seaweed-triggered inhibition to cause a transition into the heteroclinic mode when contact with the seaweed is made. This region corresponds to the ridge in the surface shown in Fig. 11a2 and the light green region in Fig. 11a1.

To better understand this middle ground, we derive an expression to approximate the mean seaweed consumption rate on the forage-and-feed task (see “Appendix 3”). This expression is derived from quantities measurable from simulations of the model performing the continuous-swallowing task: the mean bite period as a function of $\mu$ (measured when $F_\text {sw} = 0$) and the mean seaweed consumption rate as a function of $\mu$ (where all $\mu$ values are rescaled to the effective net excitation $\mu ^* = \mu (1-\kappa )$ used in the forage-and-feed task during swallowing) and $F_\text {sw}$.

Key features of the plots derived from the forage-and-feed task, including the presence and location of the maximum fitness ridge, are retained by this approximation (Fig. 11b). The differences between Fig. 11a, b can be at least partially attributed to the fact that our very simple approximation assumes that transitions from one oscillatory mode to the other are instantaneous; this is clearly not the case (e.g., see Fig. 10).

## Discussion

We have proposed a conceptual framework for understanding how motor systems can reconcile the dual requirements of robustness and flexibility, by exploiting a dynamical architecture with multiple stable modes and incorporating sensory inputs. To illustrate these concepts, we have developed and analyzed a minimal model of the *Aplysia* feeding system. We have shown that this model is bistable and can operate in two distinct oscillatory modes. Furthermore, we have shown that there are advantages and disadvantages to each mode with respect to their performance on different types of tasks, and the extent to which they are robust to different types of perturbations. In the limit cycle mode, the system is robust to perturbations to sensory input parameters, but not to perturbations in load. Furthermore, in this mode, the system is well suited for biting but not swallowing. In contrast, in the heteroclinic mode, the system is not robust to perturbations of the sensory input parameters, but is robust to perturbations in load. This mode is well suited for swallowing, but not for biting. Finally, we have demonstrated how the system can exploit the bistability between these two modes, as a way of behaving flexibly in the face of changing task demands.

Although our model is capable of oscillatory mode switching triggered solely by an applied load (Fig. 10), it is unlikely that real biological systems rely solely on this mechanism. Producing the switching behavior in the model without seaweed-triggered inhibition ($\kappa = 0$) requires careful tuning of the parameters, making the switching somewhat fragile. Moreover, it is unlikely that real *Aplysia* rely solely on proprioceptive feedback about the grasper position for detecting the presence of seaweed, since the buccal mass and surrounding tissues contain a number of mechanical and chemical sensing mechanisms (Borovikov et al. 2000; Rosen et al. 2000; Cropper et al. 2004). These reasons motivated the inclusion of seaweed-triggered inhibition in the model, which stabilized the switching behavior across a greater range of parameters (Fig. 11).

### Experimental tests of the theory

One way to test the validity of our model is to study how the neural and mechanical behaviors of *Aplysia* change in response to changing mechanical loads during feeding. Our model predicts that *Aplysia* will adjust the durations of specific motor phases in a continuous fashion for small increases in load, but may show more dramatic changes in their patterns of neural and mechanical activity, such as switching between distinct dynamical modes, when changes in load are more drastic or sudden. Prior in vivo behavioral work indicates that intact *Aplysia* switch strategies when adapting to mechanical loads of different magnitudes during feeding (Hurwitz and Susswein 1992). For low to moderate loads, feeding *Aplysia* increases their interswallow interval durations, but for high loads, *Aplysia* responds by cutting or releasing food (Hurwitz and Susswein 1992). Recently, we have shown through in vivo behavioral experiments that *Aplysia* responds to static perturbations to the load (holding the seaweed fixed) by specifically increasing the duration of the retraction phase of swallowing (Shaw et al. 2015).

Experimental tests of the model will require the appropriate biological interpretation of the control parameter $\mu$. This parameter controls the level of excitation in the three neural pools, which plays a critical role in determining the oscillatory mode of the system and the resulting behavior. Many factors can affect excitability in the neurons responsible for feeding behaviors in *Aplysia*, including internal noise within the network, and the presence or absence of neuromodulators that reflect the animal’s state of arousal. Therefore, one strategy for experimentally testing the predictions of the model would be to manipulate these variables and determine their effects on behavior and patterns of neural activity (e.g., by changing temperature in the case of intrinsic noise, or applying neuromodulators to the ganglia).

Additional tests of the model may involve recording the activity of key buccal nerves and muscles in response to systematically increasing mechanical loads during feeding. Recent advances in experimental techniques present an opportunity for testing the effects of mechanical loading on the neural dynamics, through the simultaneous recording of key nerves during behavior, either in vivo or in a semi-intact preparation (McManus et al. 2012; Cullins and Chiel 2010).

### Robustness through flexibility

The distinction between robustness and flexibility depends on the spatial and temporal scale of the behavior, as well as on how fitness is measured. Flexibility at a fine level of description (very specific fitness measures) can facilitate robustness at a more coarse-grained level of description (more general fitness measures). For example, in our model, lengthening retraction duration can be interpreted as a flexible response at the level of individual motor pools, which in turn supports robustness of swallowing overall. Similarly, switching between swallowing and biting is an example of flexibility at the level of specific tasks (biting vs. swallowing), which supports robustness of feeding more generally. Thus, the system achieves *robustness through flexibility*.

This view of robustness and flexibility as complementary rather than contradictory properties of biological systems parallels a position put forth by Lesne (2008). Lesne points out that the adaptability of biological processes at one level of description (e.g., cells and tissues) can contribute to the robustness of processes at a higher level of description (e.g., individual organisms) and that this robustness can in turn contribute to adaptability at an even higher of description (e.g., populations) (Lesne 2008). This “nested” concept of robustness and adaptability in biological systems provides a particularly useful conceptual framework for understanding *Aplysia*, since it is experimentally feasible to investigate processes at multiple spatial and temporal scales, from the level of cells and synapses up to the interaction of the entire animal with its environment (Kandel 2009; Nargeot et al. 2007; Lu et al. 2015).

### Applications to other systems

Biological systems, from the level of intracellular protein networks to evolving populations, must strike a balance between two seemingly contradictory demands: they must maintain stable patterns of activity despite noise and perturbations (robustness), but must also be able to adjust to changing environmental conditions by pursuing alternate strategies (flexibility). Mechanisms for achieving this balance have been studied in a number of contexts other than neuroscience, including genetic and metabolic networks, developmental biology, circadian rhythms, cancer biology, ecology, and population genetics (Lesne 2008; Kitano 2004; Meir et al. 2002; Akman et al. 2010; Kitano 2007; Hodgson et al. 2015; Gerhart et al. 1997; Hartwell et al. 1999).

Mathematical analysis within a dynamical systems framework (cf. “Appendix 1”) allows lessons learned to apply across domains. Nijhout et al. (2004) showed that allosteric inhibition by folate substrates results in robustness of reaction velocities (a measure of “fitness” of the folate cycle) as total folate is reduced (representing a “challenge” to the folate cycle, in the sense used in “Appendix 1”). The mathematical analysis of robust homeostasis in the folate cycle involves identifying dynamical architectures for which the sensitivity of the output of the system is small within a relevant range of parameters (Nijhout and Reed 2014; Golubitsky and Stewart 2016). Related mechanisms have been mathematically analyzed in systems responsible for regulation of dopamine synthesis (Best et al. 2009), glutathione regulation (Reed et al. 2008), and other metabolic systems (Reed et al. 2010). At the same time, robust homeostasis can reduce phenotypic variability, potentially slowing evolution (Nijhout et al. 2014). The extensive range of contexts in which these problems arise underscores the importance of developing theoretical frameworks for understanding robustness and flexibility.

Multiphasic motor patterns, and triphasic patterns in particular, are common among a wide range of motor systems. The triphasic rhythm generated by stomatogastric ganglion system is one well-studied example (Marder and Bucher 2007). Triphasic patterns are also found in vertebrate respiration (Marder 2000; Rubin et al. 2009), the cough response of mammals (Wang et al. 2009), and elsewhere (Smith et al. 1991).

Different systems can exploit either robustness or flexibility differently in response to similar types of perturbations. For example, a centipede will respond differently to the loss of a limb than a cockroach. Because of the redundancy built into the centipede’s biomechanics, it can continue to use the same gait pattern during locomotion, with only small losses in efficiency and speed (Hoffman and Wood 2013). Thus, we say that the centipede is *robust* to the loss of a limb. Cockroaches can also continue to walk after the removal of a limb, but to do so, they must adopt a new gait pattern (Delcomyn 1971). Therefore, we say that a cockroach responds *flexibly* to injury, because it can deploy an alternative strategy (a new gait) that allows it to increase its performance relative to how it would have performed had it continued to attempt to walk with the original gait. “Appendix 1” revisits the distinctions between robustness, flexibility, and sensitivity, in a dynamical systems context.

Although a general analysis of trade-offs between flexibility and robustness in biological systems may not be possible, control theory provides a promising framework (Zhou and Doyle 1998; Sontag 2013; Cowan et al. 2014). Applications of control theory to biological systems have multiplied in recent years (Lenhart and Workman 2007; Todorov and Jordan 2002; Mitchinson et al. 2007), particularly in connection with systems biology and synthetic biology (Sontag 2004; Iglesias and Ingalls 2010; Cury and Baldissera 2013; Del Vecchio and Murray 2014). But control theoretical analysis is complicated by the presence of nonlinearities, nonsmooth bifurcations, nonstationarities, and stochastic effects ubiquitous in biological control systems (Davis 1984; Isidori 1995; Åström 2012).

Here we focus on a minimal model of the *Aplysia* feeding system as an illustration of these concepts in the specific context of motor control, but it is likely that they will be applicable across a range of both biological and engineered motor systems. For example, biologically inspired robots that incorporate an SHC-based control strategy have been developed, and would be a natural setting in which to apply the concepts and analyses put forth here (Boxerbaum et al. 2012; Daltorio et al. 2013; Horchler et al. 2015).

It is important to note that studying the dynamics of the isolated nervous system alone, either theoretically or empirically, will not be sufficient for understanding the mechanisms of robustness and flexibility in motor behaviors. The biomechanics of the body, sensory feedback from the external environment, and the bidirectional interactions between the nervous system and the periphery must also be incorporated into both models and experiments to develop a more complete understanding of robustness and flexibility (Chiel and Beer 1997; Beer 1995; Chiel et al. 2009; Ting et al. 2015). Our model of the *Aplysia* feeding system is one concrete demonstration of the importance of studying coupled brain-body-environment systems rather than isolated nervous systems. We wish to emphasize that this modeling approach, as well as our theoretical framework for studying robustness and flexibility, can be applied to a wide variety of motor systems.

### Relationship to previous work

It has been previously proposed that multifunctionality in motor systems may arise from the coexistence of multiple stable attractors in the dynamics of pattern-generating circuits (Golubitsky et al. 1999; Briggman and Kristan 2008; Jing and Weiss 2001; Schwabedal et al. 2014). Other authors have pointed out that multistability in the mechanics of the body can also play a crucial role in multifunctional motor systems. For example, in the context of *Aplysia* feeding behavior, multistability in the kinematics of the feeding apparatus (the buccal mass) plays an important role in multifunctionality (Sutton et al. 2004b; Neustadter et al. 2007; Ye et al. 2006).

Our study, along with recent work by others, highlights the importance of sensory feedback in multifunctional motor systems. Toth et al. have investigated sensory feedback-triggered transitions between stepping patterns in a neuromechanical model of stick insect walking driven by a multistable CPG (Toth et al. 2012). Sensory-driven transitions between behaviors in coupled neuromechanical systems have also been studied in models of cockroach locomotion (Szczecinski et al. 2014), and in the control of locomotion in a biologically inspired robot (Haynes et al. 2012). More recently, Snyder and Rubin analyzed input-driven transitions between patterns in a multistable model of the turtle scratching CPG (Snyder and Rubin 2015).

We model the central circuit as a set of three neural pools of mutually exciting neurons with inhibitory connections between the pools. On the one hand, this structure is consistent with networks of identified neurons involved in the generation of feeding patterns in the *Aplysia* buccal ganglion Kabotyanski et al. (1993). On the other hand, our implementation takes the form of a modified version of the Lotka–Volterra equations (Lotka 1920; Volterra 1926) with three competing species. The possibility of heteroclinic cycling in such systems was described in May and Leonard (1975), who note that a general result by Smale had demonstrated that these systems could in fact produce arbitrarily complicated dynamics (Smale 1976).

The capacity of Lotka–Volterra type systems to show arbitrarily complex behavior has made them attractive for modeling complex neural firing patterns. Such Lotka–Volterra type systems with heteroclinic cycling have been used extensively as models for neural systems with sustained irregular transient activity patterns, for instance as observed during olfaction-driven oscillations (Rabinovich et al. 2001). In this paper the authors point out that a coupled system of *N* Lotka–Volterra units can in principle support up to $C_N=\sum _{k=3}^N{N\atopwithdelims ()k}(k-1)!$ distinct heteroclinic orbits.

In Levi et al. (2004), the diversity of dynamical behaviors possible in heteroclinic systems is exploited to model the chaotic movements produced by a molluskan statocyst network. As in our model, the system can exhibit different patterns of motor activity as the level of excitation varies. In our model, the endogenous excitation parameter $\mu$ controls the switch between sensitive (small $\mu$, orbits are near-heteroclinic and encounter the zero-activation boundary) and insensitive (larger $\mu$, orbits are far from the heteroclinic path and avoid the zero-activation boundary) conditions. In the statocyst model, excitation from a “hunting neuron” triggers the onset of complex, chaotic, undirected heteroclinic cycling that could help the animal hunt more effectively for unseen prey. The roles of the heteroclinic orbits are fundamentally different. In our model, heteroclinic cycling facilitates heightened sensitivity to sensory feedback, guiding the timing of behavior more effectively than in the nonheteroclinic state; in the study of *Clione* hunting, the heteroclinic trajectories permit the creature to exploit chaotic dynamics to produce complicated motor outputs. These complex movements facilitate hunting for unseen prey, but are not regulated by sensory feedback, in contrast to our model for control of feeding in *Aplysia*.

Another important difference between these two models concerns the limited number of heteroclinic orbits that are possible in our system. In our model of the *Aplysia* feeding circuit, we have $N=3$ pools, rather than an arbitrary (larger) number of interacting units. For $N=3$, there are $C_3=2$ distinct heteroclinic cycles. These two sequences correspond to two specific biologically occurring motor patterns: ingestion (the control of which we study in this paper) and egestion (which lies beyond the scope of this paper). Egestion is the attempt to expel food or other material from the buccal cavity, for instance when it is recognized as being noxious or too stiff to swallow; during egestion the animal reverses the sequence of muscle activation used during ingestion (Novakovic et al. 2006). The selection of antiphasic motor patterns—ingestion versus egestion—and the transitions between the two, which presumably involve interesting bifurcation phenomena, may be an excellent topic for a future combined modeling and experimental study.

## Appendix 1: Formal definitions of robustness and flexibility

In Sect. 1 we informally defined “robustness of a motor system with respect to a perturbation of its state variables or internal parameters” as the ability of the motor system “to maintain its fitness in performing a task in the presence of the perturbation”; in contrast we defined “flexibility” as the ability of a motor system “to deploy alternative strategies in order to perform better on different tasks or to respond to changes in the task requirements.” In this appendix we formalize these notions within a deterministic dynamical systems framework and indicate how the formally defined robustness and flexibility correspond to features of the model investigated in the main text.

By “motor system” we will mean a system of differential equations [or differential inclusions (Filippov 1988)] of the form $\frac{\mathrm{d}}{\mathrm{d}t} (\mathbf a , \mathbf x ) = \mathbf F (\mathbf a ,\mathbf x ,{\varTheta })$, where $\mathbf a$ and $\mathbf x$ are the “neural” and “body” state variables, and ${\varTheta }$ is a vector of parameters including, for example, the endogenous excitation $\mu$, the applied load $F_\text {sw}$, and the strength of sensory feedback, $\epsilon$. The vector field $\mathbf F$ may include discontinuities at a finite set of surfaces (e.g., the surface ${\varSigma }_\text {r} = \{ a_1 + a_2 = 0.5 \}$, where the grasper opens and closes, and the surfaces ${\varSigma }_{i} = \{ a_i = 0 \}$ and ${\varSigma }^{'}_{i} = \{ a_i = 1 \}$ forming the neural activation domain boundaries). Given the parameters ${\varTheta }$ and an initial condition $( \mathbf a _{0} , \mathbf x _{0})$, we have a resulting trajectory $\{ \mathbf a (t), \mathbf x (t) \}^{T}_{t =0}$ up to time *T*. We assume that for any initial condition and fixed parameters there is a bounded attractor ${\varGamma }$ (which could be a fixed point, a limit cycle, or another set with an invariant measure; for repetitive motor patterns we will assume ${\varGamma }$ is a periodic orbit). If the motor system exhibits multistability, there may be a finite collection of distinct attractors, ${\varGamma }_{i} ({\varTheta })$, for each set of parameters. Each attractor represents a “motor strategy” available to the system for parameter set ${\varTheta }$. For example, Fig. 3 shows the coexistence of the “heteroclinic mode” limit cycle attractor and the “limit cycle mode” limit cycle attractor for the neuromechanical *Aplysia* feeding model. The coexistence of multiple attractors corresponds to the coexistence of multiple “motor strategies.” We assume the boundaries between different attractors are smooth [i.e., we do not consider riddled basins of attraction (Alexander et al. 1992)].

Given a measure of fitness $S[ \{ \mathbf a (t), \mathbf x (t) \}^{T}_{t=0} ]$ for a finite trajectory, we define the fitness of the $i\text {th}$ strategy as $S_{i}[{\varTheta }] = \text {lim}_{T \rightarrow \infty } S[ \{ \mathbf a (t), \mathbf x (t) \}^{T}_{t=0} ]$ for any trajectory converging to ${\varGamma }_{i} ({\varTheta })$. We restrict fitness measures to those remaining bounded in this limit (e.g., the average rate of seaweed intake rather than the total amount of seaweed ingested). The set of strategies may gain or lose elements as ${\varTheta }$ varies, as the system crosses bifurcation boundaries separating, e.g., multistable from monostable regions. For example, it is evident from Fig. 5 that our neuromechanical *Aplysia* feeding model has regions of both bistability and monostability, in that some parameter combinations lead to all initial conditions falling into the basin of attraction of the heteroclinic mode; other parameter combinations lead to all initial conditions falling into the limit cycle mode, and other parameter sets lead to coexistence of two stable modes.

Given this dynamical framework, we may now define flexibility and robustness.

*Robustness* Consider a motor system performing a repetitive task under a reference parameter set ${\varTheta }_{0}$ using the best available strategy, ${\varGamma }_{0}({\varTheta }_{0})$, resulting in fitness $S_{0}[{\varTheta }_{0}]$. A shift in parameters ${\varTheta }_{0} \rightarrow {\varTheta }_{0} + \Delta {\varTheta }$ for which $S_{0}[{\varTheta }_{0} + \Delta {\varTheta }] > S_{0}[{\varTheta }_{0}]$ (e.g., increasing the muscle strength parameter) is an *assist*; a shift in parameters for which $S_{0}[{\varTheta }_{0} + \Delta {\varTheta }] \le S_{0}[{\varTheta }_{0}]$ (e.g., increasing the applied load) is a *challenge*. Generically, the attractors ${\varGamma }_{i}({\varTheta }_{0})$ will shift smoothly (as a point set) under sufficiently small changes in parameters, ${\varGamma }_{i} ({\varTheta }_{0}) \rightarrow {\varGamma }_{i}({\varTheta }_{0} + \Delta {\varTheta })$, leading to a small change in fitness, $S_{0}[{\varTheta }_{0} + \Delta {\varTheta }] \approx S_{0}[{\varTheta }_{0}] + \mathbf D S_{0}[{\varTheta }_{0}] \cdot \Delta {\varTheta }$ (as $\Delta {\varTheta }\rightarrow 0$). The *robustness* of a strategy ${\varGamma }_{i}$ with respect to a challenge $\Delta {\varTheta }$ is thus the component of the directional derivative $\mathbf D S_{0}[{\varTheta }_{0}]$ in the direction of the challenge, i.e., $\Delta {\varTheta }\cdot (\partial S_0/\partial {\varTheta })$. If two motor systems were challenged with the same small load, the one exhibiting a smaller local reduction in fitness would be the more robust. For example, Figs. 8 and 9 show the response of the neuromechanical *Aplysia* feeding model in the heteroclinic mode to shifts in the applied load (an increase in load being a “challenge”; a decrease in applied load is an “assist”). In the heteroclinic mode, a 40% increase in load led to only a 1% decrease in rate of seaweed intake, i.e., $(S_0[{\varTheta }_0+\Delta {\varTheta }] - S_0[{\varTheta }_0]) / S_0[{\varTheta }_0] \approx -0.01$. In contrast, under the same change in load, the fitness for the tuned limit cycle decreased by 30% (not shown), indicating that the heteroclinic mode is more robust than the limit cycle mode, at least for these particular parameters. From a mathematical point of view, our interpretation of “robustness” is similar to the approximate invariance framework introduced to characterize homeostasis (Golubitsky and Stewart 2016; Nijhout and Reed 2014).

*Flexibility* *Flexibility* refers to the coexistence of multiple attractors ${\varGamma }_{i}$ for a given parameter set ${\varTheta }$. As a challenge is increased, the fitness of the initial strategy, ${\varGamma }_{0}$ may be progressively degraded. For a sufficiently large challenge, the attractor may disappear. For example, in the neuromechanical *Aplysia* model two stable attractors (limit cycles) coexist for endogenous excitation parameter $\mu = 10^{-5}$ and applied load $F_\text {sw} = 0$ (Fig. 5); one makes contact with a hard boundary (at both $a_{0} = 0$ and $a_{1} = 0$) and the other does not. As the force is increased, the second trajectory is driven closer to the boundary until the two trajectories meet in a contact bifurcation, and the smooth limit cycle solution disappears. Coexistence of multiple attractors is a necessary but not sufficient condition for a motor system to show flexibility. To exhibit flexibility, the maximal achievable fitness, $S({\varTheta }) = \text {max}_{i} S_{i}({\varTheta })$, should exceed each individual strategy’s fitness $S_{i}({\varTheta })$ for some parameter values ${\varTheta }$. That is, no *one* strategy should have the highest fitness for all parameter values. When the neuromechanical *Aplysia* model is performing the continuous-swallowing task (Fig. 3), the heteroclinic mode outperforms the untuned limit cycle mode for all applied seaweed loads examined; for this task, the system is not flexible, since one strategy always has the highest fitness. In contrast, in the forage-and-feed task (Fig. 11), fitness is maximized when the motor system switches to the limit cycle mode when seaweed is absent from the mouth (because biting rate is maximized this way) and to the heteroclinic mode when seaweed is present (because swallowing is efficient across force levels in this mode). Thus, the model system exhibits increased performance through flexibility in the forage-and-feed task, since the motor system has access to multiple strategies, corresponding to different stable attractors, and is able to increase its fitness by switching between strategies during the task.

*Multivariate sensitivity analysis* Above, we defined the robustness of a motor system for a particular fitness function *S*, with respect to a particular challenge $\Delta {\varTheta }$, in terms of the component of $\partial S/\partial {\varTheta }$ in the direction of the challenge. In order to disentangle the multiple factors influencing this local robustness, one can consider a perturbative analysis. Formally, for small changes in load, a version of the chain rule serves as a heuristic: to find $\partial S/\partial {\varTheta }$ one must take into account the effects of changing ${\varTheta }$ on the entire limit cycle trajectory ${\varGamma }$:

18 $$\begin{aligned} \frac{\partial S}{\partial {\varTheta }}= \int _{\gamma \in {\varGamma }} \frac{\partial S}{\partial {\varGamma }}\frac{\partial {\varGamma }}{\partial {\varTheta }} \mathrm{d}\gamma . \end{aligned}$$

Here $\partial S/\partial {\varGamma }$ represents the change in the fitness measure with respect to an infinitesimal perturbation, of both the timing and the path, of the limit cycle; $\partial {\varGamma }/\partial {\varTheta }$ represents the variation in the path and timing of the limit cycle upon an infinitesimal perturbation of the parameter(s) ${\varTheta }$; and $\gamma$ represents an index, such as phase or arc length, with respect to which one integrates the effects of the load around the limit cycle.

Application of the heuristic (18) is complicated by the fact that the vector field $\mathbf {F}$ is not differentiable at all points along the limit cycle ${\varGamma }$, for instance at the sections of discontinuity ${\varSigma }_r,{\varSigma }_i$ (above).^9^ A complete analysis lies beyond the scope of the present paper. As a first step, we may consider the accumulated effect for each segment of the trajectory between successive surfaces of continuity. That is, we may break up the integral (18) into a sum of contributions from each segment of the limit cycle lying within a domain within which $\mathbf {F}$ is smooth.

The dynamical systems framework presented here allows us to make precise interpretations of the heuristic definitions of robustness, flexibility, and sensitivity introduced in Sect. 1.1. In Sect. 2.7 of the main text, we apply these general notions to the specific neuromechanical model.

**Table 1:** Model parameters

Parameter	Value	Description
$\gamma$	2.4	Inhibition strength from next pool
$\epsilon$	0.002	Sensory feedback strength
$\kappa$	0.5	Strength of the load-sensing inhibition
$\mu$	$10^{-5}$	Neural pool intrinsic excitation
$\tau _{a}$	0.05	Neural pool time constant
$\tau _{m}$	2.45	Muscle activation time constant
$b_{\mathrm{r}}$	0.4	Grasper damping constant
$c_{0}$	1.0	Position of shortest length for I2
$c_{1}$	1.1	Position of center of I3
$F_{\text {sw}}$	0.01	Force on the seaweed resisting ingestion
$\sigma _{0}$	−1	Sign of proproceptive input to $a_0$ neural pool
$\sigma _{1}$	1	Sign of proproceptive input to $a_1$ neural pool
$\sigma _{2}$	1	Sign of proproceptive input to $a_2$ neural pool
$S_{0}$	0.5	Proprioceptive neutral position for protraction-open neural pool
$S_{1}$	0.5	Proprioceptive neutral position for protraction-closed neural pool
$S_{2}$	0.25	Proprioceptive neutral position for retraction-closed neural pool
$u_{\text {max}}$	1.0	Maximum muscle activation
$w_{0}$	2	Maximal effective length of I2
$w_{1}$	1.1	Maximal effective length of I3

**Table 2:** State variables

State variable	Initial value	Description
$a_{0}$	$1 - 10^{-9}$	Activity of protraction-open neural pool (non-negative)
$a_{1}$	$10^{-9}$	Activity of protraction-closing neural pool (non-negative)
$a_{2}$	$10^{-9}$	Activity of retraction-closed neural pool (non-negative)
$u_{0}$	0	Activity of I2 muscle
$u_{1}$	0	Activity of I3 muscle
$x_{r}$	0.5	Grasper position (0 is retracted, 1 is protracted)
$x_{\text {sw}}$	0	Seaweed position (positive is away from the animal)

## Appendix 2: Parameters and state variables

Table 1 lists interpretations and default values of all parameters for both the heteroclinic mode and the untuned limit cycle mode simulations. The tuned limit cycle mode simulations use these parameters as well, with the exception of those parameters described in “Tuned limit cycle mode parameters” section.

Table 2 lists the initial conditions used for the heteroclinic mode and tuned limit cycle mode simulations. For the untuned limit cycle mode simulations, these are modified so that the neural variables have initial values (0.2, 0.4, 0.7).

### Tuned limit cycle mode parameters

**Table 3:** Parameters used for the limit cycle simulations

Parameter	Value	Description
$\mu$	$10^{-4}$	Neural pool intrinsic excitation
$\beta$	0.143	Neural pool global time constant
$\alpha _{0}$	0.61	Neural pool local time scaling near protraction-open
$\alpha _{1}$	$-0.92$	Neural pool local time scaling near protraction-closed
$\alpha _{2}$	0.277	Neural pool local time scaling near retraction-closed

For comparing the robustness of the two oscillatory modes in the continuous-swallowing task, we tuned the parameters of the limit cycle mode so that each phase of the neural activation sequence of the two modes had comparable durations, and so that the two modes consumed seaweed at approximately the same rate. Specifically, we chose a value of $\mu$ sufficiently large that perturbing the other parameters would not cause the system to switch into the heteroclinic mode. We also asymmetrically adjusted the rate of cycling of the neural variables by replacing the uniform neural time constant $\tau _{\text {a}}$ with the following phase-dependent time scaling function:

19 $$\begin{aligned} \tau _\text {a}(\mathbf {a}) = (1 + \alpha \cdot \mathbf {a}) \beta . \end{aligned}$$

Here $\beta$ is a scalar parameter that uniformly adjusts the timescale of the neural activity, and $\alpha$ is a vector parameter representing an activity-dependent scaling of the speed. This change affects the timing of the cycle, but does not significantly change the geometry of the trajectories in the three-dimensional space of the neural variables. The values of $\mu$, $\beta$, and $\alpha$ for the tuned limit cycle mode simulations are given in Table 3.

## Appendix 3: Derivation of expression approximating fitness in the forage-and-feed task

To better understand the shape of the plots in Fig. 11a1, a2, which plot the fitness of the model when it is permitted to flexibly switch between oscillatory modes (the forage-and-feed task), here we provide an expression approximating those fitness surfaces derived from quantities measured when the model is operating in only one mode.

The form of the expression depends on whether the mean intake rate for the constant load continuous-swallowing task is positive or negative. When the rate is negative (as may be the case when the model is in the limit cycle mode, e.g., Fig. 3b), we assume that the mean intake for the forage-and-feed task is zero, since the model will (on average) fail to consume any seaweed that is grasped. For values of $\mu ^*$ and $F_\text {sw}$ such that the mean intake rate for the constant load continuous-swallowing task is positive, we derive an expression via the following argument.

The mean period of a bite in the forage-and-feed task, ${\langle T_\text {B} \rangle }_{\mu }$, measured in seconds, depends on the endogenous excitation $\mu$, and we will estimate its value from empirical measurements on the continuous-swallowing task with $F_\text {sw} = 0$.

Since the transition to swallowing from biting in the forage-and-feed task can occur only at the moment the grasper closes, we assume that an integer number of bites occurs between each swallowing phase of the forage-and-feed task. This number follows a geometric distribution with mean 1 / *p*, where *p* is the probability of grasping the seaweed each bite. Thus, the mean interval between swallowing phases, measured in seconds, is

$$\begin{aligned} \frac{{\langle T_\text {B} \rangle }_{\mu }}{p}. \end{aligned}$$

The mean rate of seaweed intake during the swallowing phases of the forage-and-feed task, ${\langle S_\text {S} \rangle }_{\mu ^{*},F_\text {sw}}$, measured in seaweed units per second, depends on both the endogenous excitation $\mu ^{*}$ and the load $F_\text {sw}$. We will estimate its value as well from empirical measurements on the continuous-swallowing task.

Since each seaweed strip in the forage-and-feed task has a fixed length *L*, the mean duration of each swallowing phase, measured in seconds, is

$$\begin{aligned} \frac{L}{{\langle S_\text {S} \rangle }_{\mu ^{*},F_\text {sw}}}. \end{aligned}$$

The mean rate of seaweed intake throughout the entire forage-and-feed task, ${\langle S \rangle }_{\mu ,\mu ^{*},F_\text {sw}}$, measured in seaweed units per second, is our measure of the performance of the model on the forage-and-feed task. This is equal to the length of one seaweed strip divided by the amount of time spent grasping for (“foraging for”) and swallowing (“feeding on”) the strip:

20 $$\begin{aligned} {\langle S \rangle }_{\mu ,\mu ^*,F_\text {sw}} = {\left\{ \begin{array}{ll} \frac{L}{\frac{{\langle T_\text {B} \rangle }_{\mu }}{p} + \frac{L}{{\langle S_\text {S} \rangle }_{\mu ^*,F_\text {sw}}}} &{} {\langle S_\text {S} \rangle }_{\mu ^*,F_\text {sw}} > 0, \\ 0 &{} {\langle S_\text {S} \rangle }_{\mu ^*,F_\text {sw}} \le 0. \\ \end{array}\right. } \end{aligned}$$

Figure 11b1, b2 plots Eq. 20 using parameters matching the simulations that generated Fig. 11a1, a2. Qualitatively, there is excellent correspondence between the major features of the numerical and theoretical fitness surfaces. Quantitative discrepancies may be attributed to simplifying assumptions made during this derivation, such as the assumption of an instantaneous transition from one oscillatory mode to another.

## Footnotes

1 Here we use the term “fitness” to refer to the performance of a motor system on a specific task, not the overall success of the organism in terms of survival and reproduction.

2 Simulations indicate that both $\Delta x$ and *T* can be reasonably approximated as linear functions of load for the heteroclinic mode, the tuned limit cycle mode, and the untuned limit cycle mode over at least the range of total loads [0, 0.025].

3 The model in Shaw et al. (2015) lacks seaweed-triggered inhibition (the second term in Eq. 6). However, in that paper we explored only the continuous-swallowing task, and this term is only relevant to the forage-and-feed task. Thus, the inclusion of this term does not affect the behavior of the oscillatory modes or the existence of bistability for the continuous-swallowing task.

4 Note that the net input becoming negative for a brief period does not guarantee a collision with the boundary. If the neural trajectory is not sufficiently close to the boundary when the negative input occurs, it will miss the boundary and continue progressing through the cycle, as shown in the top plot in Fig. 4.

5 Biologically, we interpret these perturbations as changing the baseline strengths of the different sensory pathways across the range of grasper positions, which could be accomplished experimentally through lesioning or the targeted application of modulatory compounds.

6 To facilitate direct comparisons of the robustness of the two modes, we first increased the endogenous excitation $\mu$ for the limit cycle mode to $10^{-4}$ to prevent changes to the proprioceptive feedback from switching the system into the heteroclinic mode. We then tuned the time constants of the limit cycle mode so that the performance is on par with that of the heteroclinic mode (see “Tuned limit cycle mode parameters” section in “Appendix 2”).

7 In fact, the pattern is slightly shorter.

8 We observe for both oscillatory modes that the following equalities are approximately true while the system is following a stable orbit: $a_0 + a_1 + a_2 = 1$, $u_0 + u_1 = u_\text {max}$. By projecting the neural variables onto the plane $a_0 + a_1 + a_2 = 1$ (Fig. 9, left), we visualize their trajectory in two dimensions without significant loss of information. Similarly, by plotting grasper position against the quantity $u_0/u_\text {max}$ (Fig. 9, right), we visualize the approximate trajectory of the mechanical variables, where values close to 1 of the latter quantity correspond to activation of the protractor muscle, and values close to 0 to activation of the retractor muscle.
